# Enzymatic Enantioselective Decarboxylative Protonation of Heteroaryl Malonates

**DOI:** 10.1002/chem.201406014

**Published:** 2015-03-12

**Authors:** Ross Lewin, Mark Goodall, Mark L Thompson, James Leigh, Michael Breuer, Kai Baldenius, Jason Micklefield

**Affiliations:** [a] School of Chemistry and Manchester Institute of Biotechnology, The University of Manchester 131 Princess Street, Manchester M1 7ND (UK) E-mail: jason.micklefield@manchester.ac.uk; [b] BASF SE, GVF/E 67056 Ludwigshafen (Germany)

**Keywords:** biocatalysis, decarboxylase, hydroxycarboxylic acids, enzyme mechanism, synthesis

## Abstract

The enzyme aryl/alkenyl malonate decarboxylase (AMDase) catalyses the enantioselective decarboxylative protonation (EDP) of a range of disubstituted malonic acids to give homochiral carboxylic acids that are valuable synthetic intermediates. AMDase exhibits a number of advantages over the non-enzymatic EDP methods developed to date including higher enantioselectivity and more environmentally benign reaction conditions. In this report, AMDase and engineered variants have been used to produce a range of enantioenriched heteroaromatic α-hydroxycarboxylic acids, including pharmaceutical precursors, from readily accessible α-hydroxymalonates. The enzymatic method described here represents an improvement upon existing synthetic chemistry methods that have been used to produce similar compounds. The relationship between the structural features of these new substrates and the kinetics associated with their enzymatic decarboxylation is explored, which offers further insight into the mechanism of AMDase.

## Introduction

**Figure 1:**
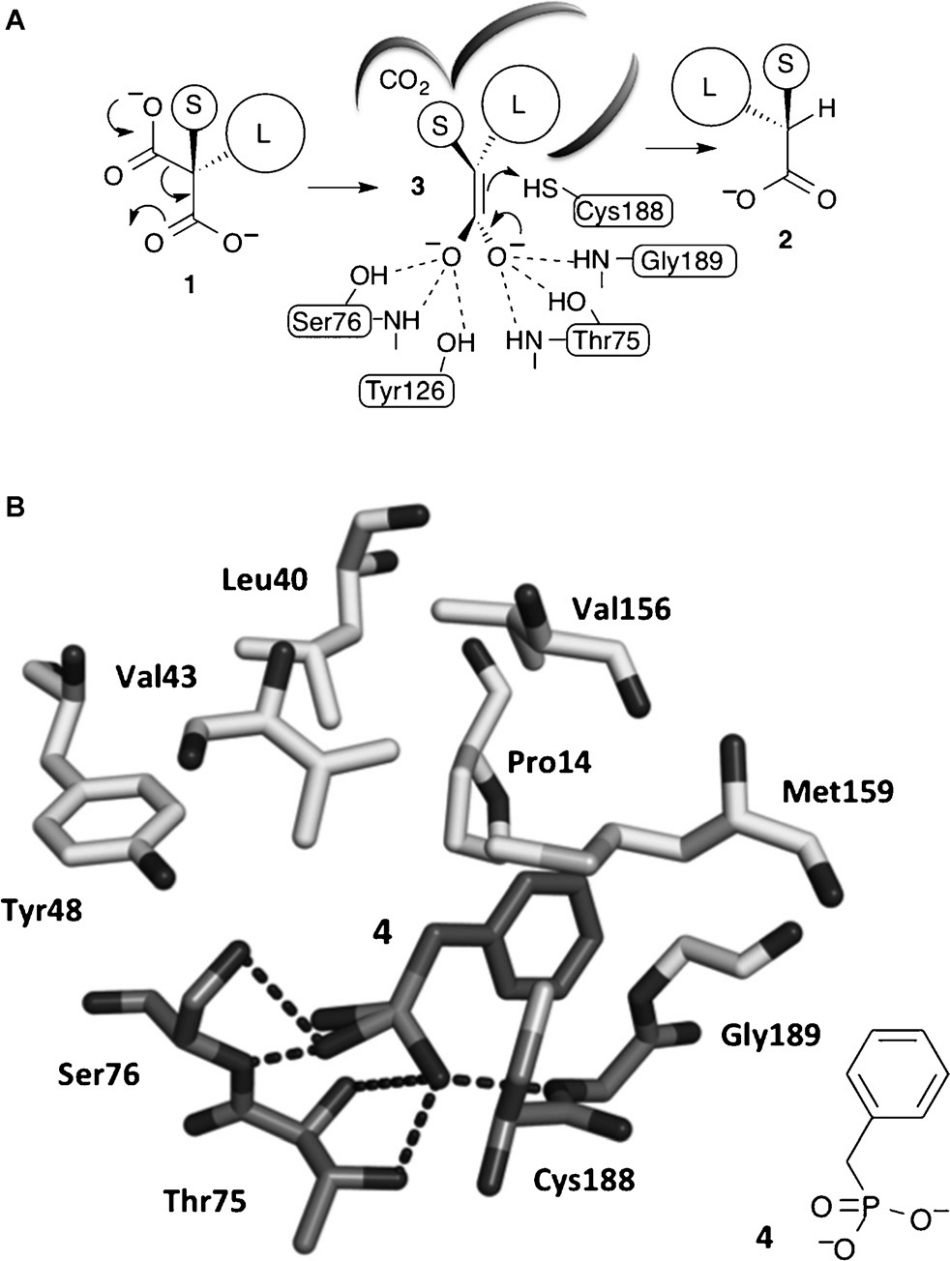
A) The proposed mechanism of AMDase-catalysed decarboxylation reactions. B) X-ray crystal structure of AMDase with a benzyl phosphonate inhibitor 4 bound in the active site.[2] Hydrogen bonding between the dianion and the residues that make up the “dioxyanion hole” is shown in darker grey. Tyr126 is removed for clarity.

The aryl/alkenyl malonate decarboxylase (AMDase) catalyses the decarboxylation of α-aryl and α-alkenyl malonic acids **1** to produce enantioenriched carboxylic acids **2** (Figure 1A).[1–7] AMDase belongs to the Asp/Glu racemase superfamily of enzymes that are widespread across bacteria and include glutamate,[8–11] aspartate[12] and hydantoin racemases[13, 14] as well as maleate isomerases.[15, 16] Although the natural metabolic function of AMDases is not known, these enzymes can effect the stereoselective decarboxylation of a range of disubstituted malonic acids **1**, provided that one α-substituent is aryl or alkenyl and the other is a small functional group, for example, methyl, hydroxy or amino.[2]

Recently we solved X-ray crystal structures of the *Bordetella bronchiseptica* AMDase (3DG9 and 3IP8).[1, 2] These were the first structures of this class of enzyme and revealed a “dioxyanion hole” motif that we suggest donates six hydrogen bonds required to stabilise a putative high-energy enediolate intermediate **3** that is formed upon decarboxylation (Figure 1A). In silico docking of an enediolate intermediate from the decarboxylation of α-methyl-α-phenyl malonic acid (**1**, L=Ph & S=Me), into the active site of the enzyme, shows how the phenyl group occupies a large solvent accessible cavity. At the opposite side of the active site is a small hydrophobic pocket that accommodates the methyl substituent, with the thiol of Cys188 placed 4.16 Å from the *Si* face of the enediolate, ideally positioned for protonation that leads to the observed *R*-configured α-phenylpropionic acid (**2**, L=Ph & S=Me) (Figure 1A). Other enzymes of the mechanistically related enolase superfamily are known to utilise Mg^2+^ as well as H-bonding to stabilise similar enediolate intermediates.[17, 18] However, the AMDase does not require metal ions or any other co-factors, which suggests that the dioxyanion hole is all that is required to stabilise the enediolate.[19] To gain further evidence to support the intermediacy of an enediolate,[19] we solved a second independent structure of the AMDase with a benzyl phosphonate inhibitor **4**, which mimics the structural and electronic properties of the enediolate, bound to the active site of the enzyme. This showed that the phosphonate **4** binds to the dioxyanion hole as predicted, with the phenyl group occupying the large solvent accessible channel (Figure 1B). ^18^O-labelling studies, also showed that the *pro*-*R* carboxylate of α-methyl-α-phenyl malonic acid (**1**, L=Ph & S=Me) is lost with overall inversion of stereochemistry and enabled the orientation of the substrate in the active site to be defined.[1] With the *pro*-*S* carboxylate bound to the dioxyanion hole, the *pro*-*R* carboxylate of the model substrate α-methyl-α-phenyl malonic acid (**1**, L=Ph & S=Me) must occupy the smaller hydrophobic pocket. In this orientation, the *pro*-*R* carboxylate is deprived of any stabilising H-bonding or other electrostatic interactions. We suggest that this destabilisation of the *pro*-*R* carboxylate triggers the decarboxylation, favouring the formation of neutral CO_2_, with delocalisation of the residual electron density into the enediolate.[19]

Based on these insights we were able to expand the biocatalytic repertoire of the AMDases showing that, whilst the enzyme does not accept α,α-dialkyl malonic acids, it will accept a wide range of α-alkenyl malonic acids with additional α-methyl, α-amino and α-hydroxy substituents.[2] It is thus essential for AMDase catalysis that malonic acid substrates possess an α-substituent with a π-system that can adopt a co-planar geometry and stabilise the resulting enediolate through additional conjugation. Furthermore, we have shown that the AMDase is also amenable to directed evolution.[2] Using iterative saturation mutagenesis, AMDases were evolved with up to 50-fold increased activity, whilst retaining excellent enantioselectivity.[2] In this study we further explore the biocatalytic potential of AMDases, and improved mutants from directed evolution, for the chemoenzymatic synthesis of a series of heteroaryl α-hydroxy acids, which include a number of key intermediates required for the synthesis of pharmaceuticals and other valuable products.

## Results and Discussion

Homochiral α-hydroxy acids are widely used intermediates in the preparation of pharmaceuticals, agrochemicals and other commercially important products. For example, α-heteroaryl α-hydroxy acids are intermediates in the preparation of the commercial antidepressant duloxetine,[20] cognitive enhancer T-588,[21] and are also key constituents of fungicides.[22] Previously, a range of α-alkenyl-α-hydroxy malonic acids[2] and α-phenyl-α-hydroxy malonic acids[23] were shown to be good substrates for the AMDases, producing α-hydroxy acids in high enantiomeric excess (*ee*). In addition, we envisaged that α-heteroaryl α-hydroxy malonic acid substrates could be conveniently prepared from the reaction of ketomalonates with heteroaromatic compounds, including heteroaryl Grignard and organolithium reagents.

**Scheme 1:**
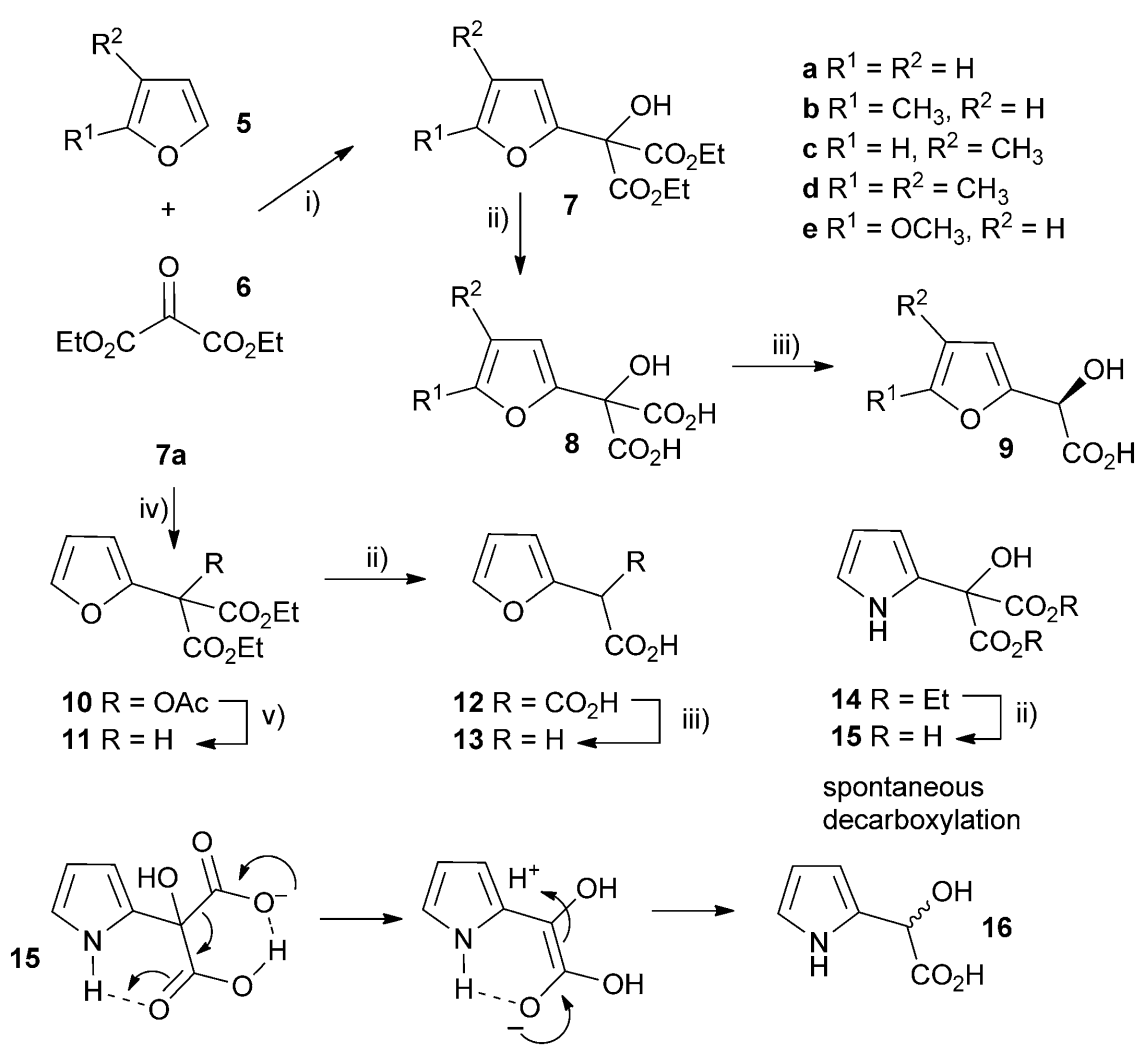
Synthesis of furan-2-yl malonic acids 8 a–e and subsequent AMDase-catalysed decarboxylation to give enantioenriched furanyl α-hydroxy acids 9 a–e. The proposed mechanism through which α-hydroxy-α-pyrrol-2-yl malonic acid 15 spontaneously decarboxylates is also shown. i) A neat equimolar mixture of 5 and 6, overnight, RT; ii) EtOH/H_2_O, NaOH (2.1 equiv), 80 °C, 3 h; iii) malonate substrate (500 mm), AMDase (0.001 mm), DTT (0.05 mm) TRIS buffer (10 mm, pH 7.0); iv) Ac_2_O (10 equiv), Et_3_N (3 equiv), DMAP (0.5 equiv), dichloromethane, 48 h, RT; v) Na (30 equiv), α-(dimethylamino)naphthalene (30 equiv), DMPU, 15 h, RT. DTT=dithiothreitol; DMAP=4-dimethylaminopyridine; DMPU=1,3-dimethyl-3,4,5,6-tetrahydro-2(1*H*)-pyrimidinone.

Initially the preparation of α-furanyl-α-hydroxy acids was explored. Accordingly, furans **5 a**–**e** were treated with diethyl ketomalonate **6**, under solvent-free conditions,[24] to generate the α-furanyl-α-hydroxy diethyl malonates **7 a**–**e** in yields of ca. 45–50 % (Scheme 1). The diethyl malonates **7 a**–**e** were then hydrolysed using sodium hydroxide to form their respective malonic acids **8 a**–**e** in near quantitative yields. Subsequent decarboxylation of the malonic acids **8 a**–**e** catalysed by the wild-type *B. bronchiseptica* AMDase gave furanyl α-hydroxy carboxylic acids **9 a**–**e** in good yields. The kinetic parameters associated with the AMDase-catalysed decarboxylation of these furanyl substrates **8 a**–**e** was determined spectrophotometrically as previously described (Table 1).[1, 2] The *ee* values of the products were determined using a chiral-HPLC methodology (Table 1).

**Table 1:** Kinetic parameters associated with the decarboxylation of a wide-range of substrates by both WT and mutant AMDase enzymes^[a]^

Substrate	Enzyme	*K*_m_^[a]^ [mm]	*k*_cat_^[a]^ [s^−1^]	*k*_cat_/*K*_m_^[a]^ [s^−1^ mm^−1^]	*ee*^[b]^ [%]	Product
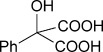	wt.	30.2±2.4	101±12	3.3±0.7	96	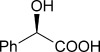
	M159V					
	P14V/P15G					
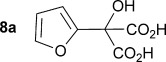	wt.	8.6±2.1	61.9±6.0	6.4±0.4	95	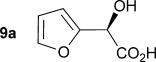
	M159V	7.2±1.8	149.3±64.1	20.7±0.7	95	
	P14V/P15G	6.9 1.6	66.4±17.4	9.6±0.5	93	
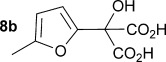	wt.	9.8±2.2	37.1±8.8	3.8±0.5	95	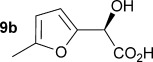
	M159V	9.5±2.2	55.9±18.1	5.9±0.6	95	
	P14V/P15G	7.3 2.3	35.9±10.6	4.9±0.6	96	
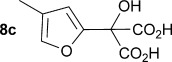	wt	13.3±4.3	42.4±15.5	3.2±0.7	95	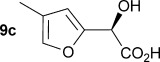
	M159V	8.9±4.5	55.9±24.6	6.3±0.9	91	
	P14V/P15G	9.9±5.2	34.0±13.0	3.4±0.9	90	
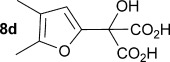	wt	12.7±3.9	12.4±3.9	1.0±0.6	96	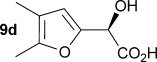
	M159V	11.4±3.5	35.0±14.0	3.1±0.7	90	
	P14V/P15G	12.6±1.6	28.8±9.0	2.3±0.9	90	
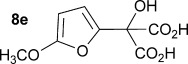	wt	16.1±4.5	30.5±9.4	1.9±0.6	84	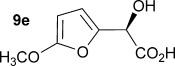
	M159V	10.7±4.0	35.0±14.0	3.2±0.7	80	
	P14V/P15G	7.2±1.1	28.8±9.0	4.1±0.4	84	
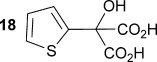	wt	4.3±0.5	165.9±10.6	38.6±0.2	97	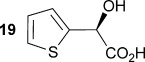
	M159V	3.5±1.3	79.3±19.0	22.4±0.6	93	
	P14V/P15G	2.2±0.5	34.0±3.5	15.2±0.3	96	
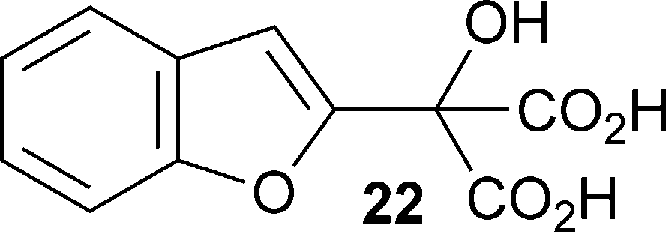	wt	8.2±1.6	277.9±33.9	33.9±0.32	93	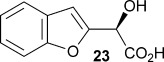
	M159V	3.6±1.1	182.1±39.4	50.6±0.52	91	
	P14V/P15G	7.4±1.8	115.3±19.7	15.6±0.41	91	
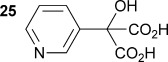	wt	6.4±0.6	224.8±36.3	35.3±0.25	97	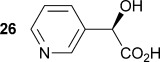
	M159V	7.3±2.8	86.4±36.3	11.8±0.8	95	
	P14V/P15G	13.0±5.7	42.2±13.6	3.2±0.7	96	
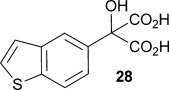	wt	3.1±0.17	147.7±4.6	48.3±0.17	89	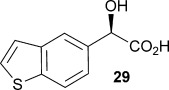
	M159V	0.82±0.11	54.8±2.1	66.6±0.17	84	
	P14V/P15G	2.1±0.3	68.5±22.1	9.9±0.2	85	

**Figure 2:**
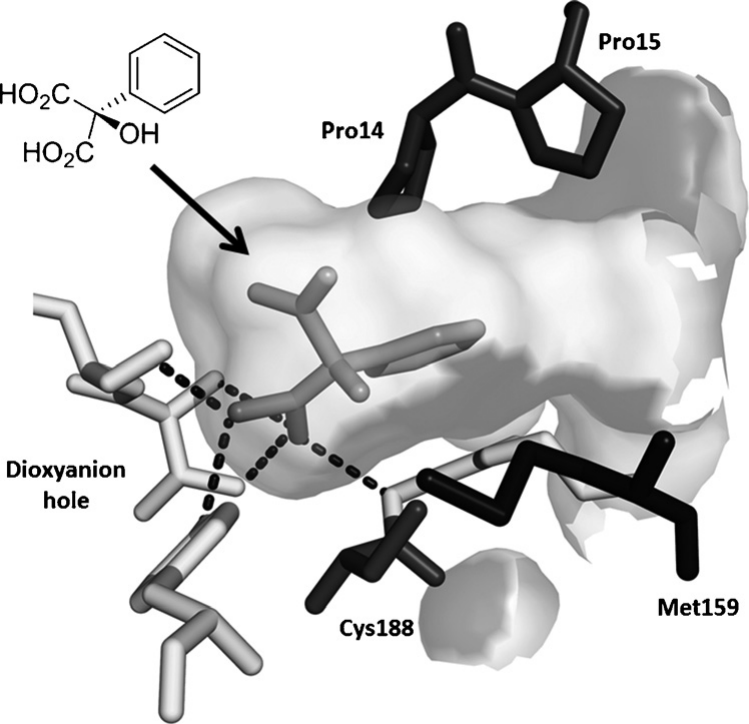
Active site of AMDase with α-hydroxy-α-phenylmalonic acid (medium grey) bound. The important residues that are highlighted are the P14, P15 and M159 (dark grey) altered during our mutagenesis studies, the catalytic C188 and residues of the dioxyanion hole (lighter grey).

[a] See the Supporting Information for the methods employed to determine kinetic parameters. [b] For some substrates the enantiomeric excess (*ee*) of the product is observed to be below 99 %; this is not due to reduced enantioselectivity of the AMDase, and mutants, rather it is a consequence of the competing non-enzymatic, spontaneous backround, decarboxylation reactions. In this respect, the *ee* values are a measure of the relative rates of enzymatic vs. non-enzymatic decarboxylation. The stereochemistry of the α-hydroxy acid products **9 a**–**e**, **19**, **23**, **26** and **29** are assigned based on: the stereochemical course of AMDase-catalysed decarboxylation reaction as determined previously by detailed labelling experiments and high resolution X-ray structures of AMDase (Figure 1);[1, 2] the configuration of many AMDase products as determined previously;[1–7] preparation of Mosher’s esters for **19** (see Figure S1 and Table S1 in the Supporting Information); comparison of CD spectra with natural (*R*)-mandelic acid and **19** (see Figures S2–S10 in the Supporting Information); and by comparison with previously determined optical rotations for compounds **23**, **26** and **29** (see the Supporting Information).

Analysis of the kinetic data (Table 1) reveals that compounds **8 a**–**e** are good substrates for the wild-type AMDase, resulting in the enantioenriched products **9 a**–**e**. Notably, compounds **8 a**–**e** possess a lower *K*_m_ value than the corresponding α-hydroxy-α-phenyl malonic acid, which is consistent with the furanyl malonates binding more tightly to the AMDase active site. On the other hand, the *k*_cat_ values for the furanyl substrates **8 a**–**e** are lower than the *k*_cat_ for α-hydroxy- α-phenylmalonate. This is consistent with the phenyl group providing more effective delocalisation of the electron density that develops during decarboxylation than the electron-rich furan substituent. The phenyl group can presumably stabilise the transition state leading to the putative enediolate to a greater extent than the furanyl group can. In previous studies α-phenyl malonic acid (**1**, L=Ph & S=H) proved to be a better substrate than α,α-disubstituted malonic acids such as α-hydroxy-α-phenyl malonic acid (**1**, L=Ph and S=OH). To test whether this is also the case for the furan series of substrates, α-(furan-2-yl) malonic acid **12** was produced (Scheme 1). Accordingly, α-(furan-2-yl)-α-hydroxy diethyl malonate **7 a** was acetylated with acetic anhydride; reduction of the acetyl derivative **10** with sodium in DMPU, in the presence of α-(dimethylamino)naphthalene, gave α-(furan-2-yl) diethyl malonate **11**; and this was subsequently hydrolysed to give the required malonic acid **12**. The resulting α-furan-2-yl malonic acid **12** proved to be a superior substrate (*K*_m_=10.1±4.5 mm, *k*_cat_=188.2±77 s^−1^ mm^−1^) compared to the disubstitued furanyl substrates **8 a**–**e**. However, the *k*_cat_ value for decarboxylation of α-(furan-2-yl) malonic acid **12** to form α-(furan-2-yl) acetic acid **13** is around half of that observed for decarboxylation of α-phenyl malonic acid (**1**, L=Ph & S=H), with both substrates exhibiting similar *K*_m_ values. This further reflects the greater stabilisation of the transition state generated during decarboxylation of malonic acids possessing a phenyl substituent compared with a furyl substituent.

In addition to furans, we also sought to explore if α-(pyrrol-2-yl) malonic acids are useful AMDase substrates. Accordingly, reaction of pyrrole with ketomalonate **6** afforded the diester **14**, which was hydrolysed to generate α-hydroxy-α-(pyrrol-2-yl) malonic acid **15**. However, **15** proved unstable and underwent rapid spontaneous decarboxylation. The spontaneous decarboxylation of **15** might be attributed to the propensity of the pyrrolic amino group of **15** to form an intramolecular H-bond with the carboxyl groups, which would facilitate spontaneous decarboxylation to give the racemic α-hydroxy-α-(pyrrol-2-yl) acetic acid **16** (Scheme 1).

In previous directed evolution experiments a single mutant M159V and double mutant P14V/P15G emerged as enzymes with improved activity for a range of alkenyl and aryl malonic acids.[2] After having established that furyl malonates **8 a**–**e** and **12** are substrates for the wild-type AMDase, the activity of these optimised AMDase mutants with **8 a**–**e** and **12** was also explored. As anticipated, the M159V mutant leads to improved *k*_cat_ values for decarboxylation of **8 a** with a *k*_cat_ for the mutant 2.4-fold higher compared with the wild-type AMDase. The *K*_m_ value remained relatively unaffected by the mutation M159V. This is in general agreement with the kinetic data obtained from alkenyl and aryl malonic acids[2] and is consistent with the suggestion that the M159V mutant provides more space around Cys188, increasing the conformational flexibility of this catalytic residue, and thereby improving interactions with the transition state from decarboxylation leading to the enediolate (Figure 2).

From analysis of the two AMDase X-ray crystal structures it is apparent that the P14V/P15G double mutant (Figure 2) creates more space in the large solvent accessible channel that accommodates the larger aromatic substituent of typical α-aryl malonate substrates. For **8 e**, the kinetic data (Table 1) indicates that these two combined mutations results in more favourable *K*_m_ values. Presumably, the increased size of the solvent accessible channel of the P14V/P15G mutant improves the binding of the larger 5-methoxyfuranyl group. Interestingly, for α-(4,5-dimethylfuran-2-yl)-α-hydroxy malonic acid **8 d** the P14V/P15G mutations have little effect on *K*_m_, but do give rise to a significant two-fold increase in *k*_cat_ relative to the wild-type enzyme. Presumably the P14V/P15G mutant allows the α-(4,5-dimethylfuran-2-yl) substituent of **8 d** to more readily adopt the co-planar orientation required to stabilise the transition state leading to the enediolate intermediate.

**Scheme 2:**
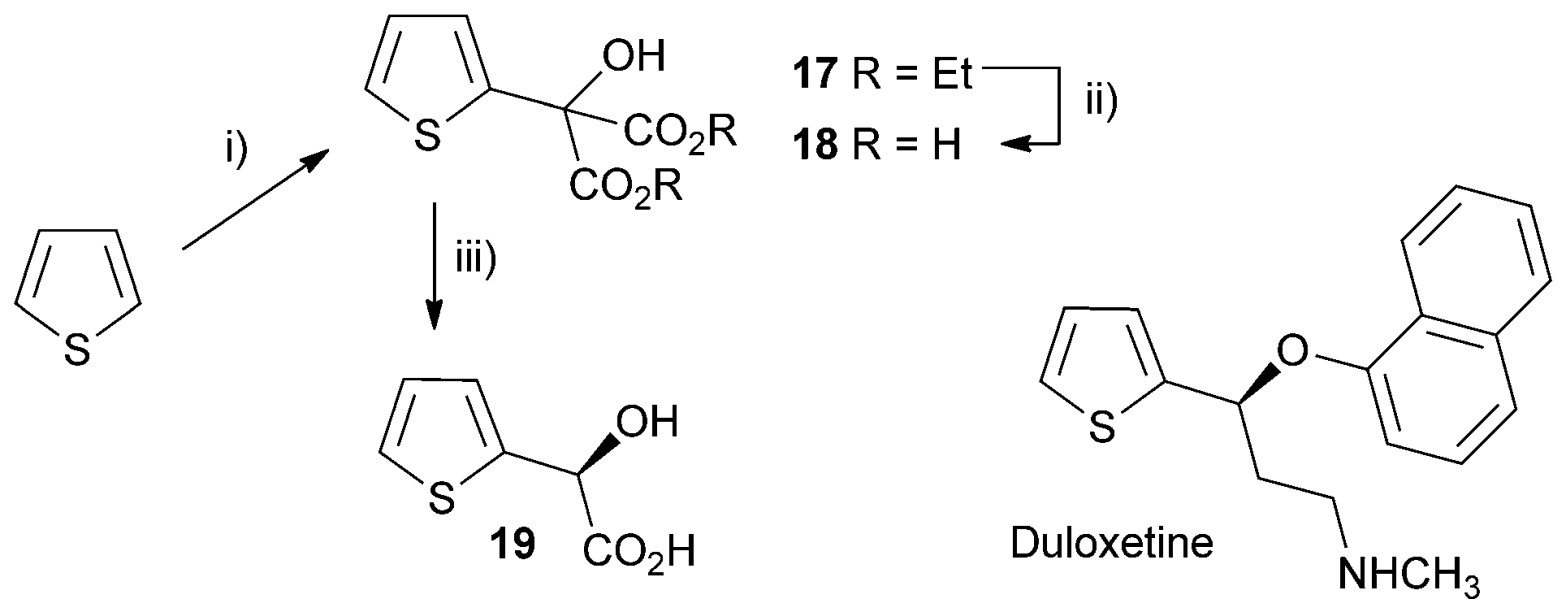
Chemoenzymatic synthesis of α-hydroxy-α-(thiophen-2-yl) acetic acid 19, a building block towards the synthesis of duloxetine. i) *n*BuLi, Et_2_O, −78 °C, then diethylketomalonate 6; ii) NaOH (2.2 equiv), EtOH/dichloromethane, 3 h, RT; iii) malonate 18 (500 mm), AMDase (0.001 mm), DTT (0.05 mm) TRIS buffer (10 mm, pH 7.0), 6 h.

To demonstrate the potential of this chemoenzymatic approach for the synthesis of commercially relevant pharmaceutical intermediates, α-hydroxy-α-(thiophen-2-yl) malonic acid **18** was prepared. It was envisaged that decarboxylation of **18** would afford α-hydroxy-α-(thiophen-2-yl) acetic acid **19** which is an established precursor in the synthesis of duloxetine, a therapeutically and commercially important serotonin-norepinephrine reuptake inhibitor (Scheme 2). In this case, thiophene required lithiation with *n*-butyl lithium to enable a subsequent nucleophilic addition to diethyl ketomalonate **6**. Hydrolysis was attempted on the resulting diester **17** by using NaOH in a mixture of ethanol and water as described previously for **7 a**–**e**, but this resulted in decarboxylation. Instead, ester hydrolysis was achieved, by using sodium hydroxide in methanol, with dichloromethane, which results in the precipitation of malonic acid **18** as a sodium salt. Malonate **18** proved to be a good substrate for the AMDase, affording the desired product **19**. The absolute configuration of **19** was confirmed through ^1^H NMR spectroscopic analysis of the corresponding Mosher ester[25] and showed that the product possessed a (2*S*)-configuration in line with the previously observed stereoselectivity of the AMDase established for alkenyl and aryl malonates[1, 2] (see the Supporting Information). Kinetic parameters obtained for **18** with the wild-type AMDase showed that this was a better substrate than its furanyl analogue **8 a** in terms of both *K*_m_ and *k*_cat_; the higher *k*_cat_ value being due to the greater degree of aromaticity associated with thiophene compared to furan. The kinetic parameters for single M159V and double P14V/P15G mutants were obtained in the expectation that these mutations would result in improved activity compared to that of the wild type. However, this was not observed, with a *k*_cat_ value for M159V that was roughly half that of the wild type, and a *k*_cat_ value for P14V/P15G that was roughly half that again. As a consequence of the lower catalytic activity of the mutant AMDase non-enzymatic background decarboxylation competes with the enzymatic decarboxylation resulting in slightly less than perfect *ee* values for the product **19** (Table 1).

**Scheme 3:**
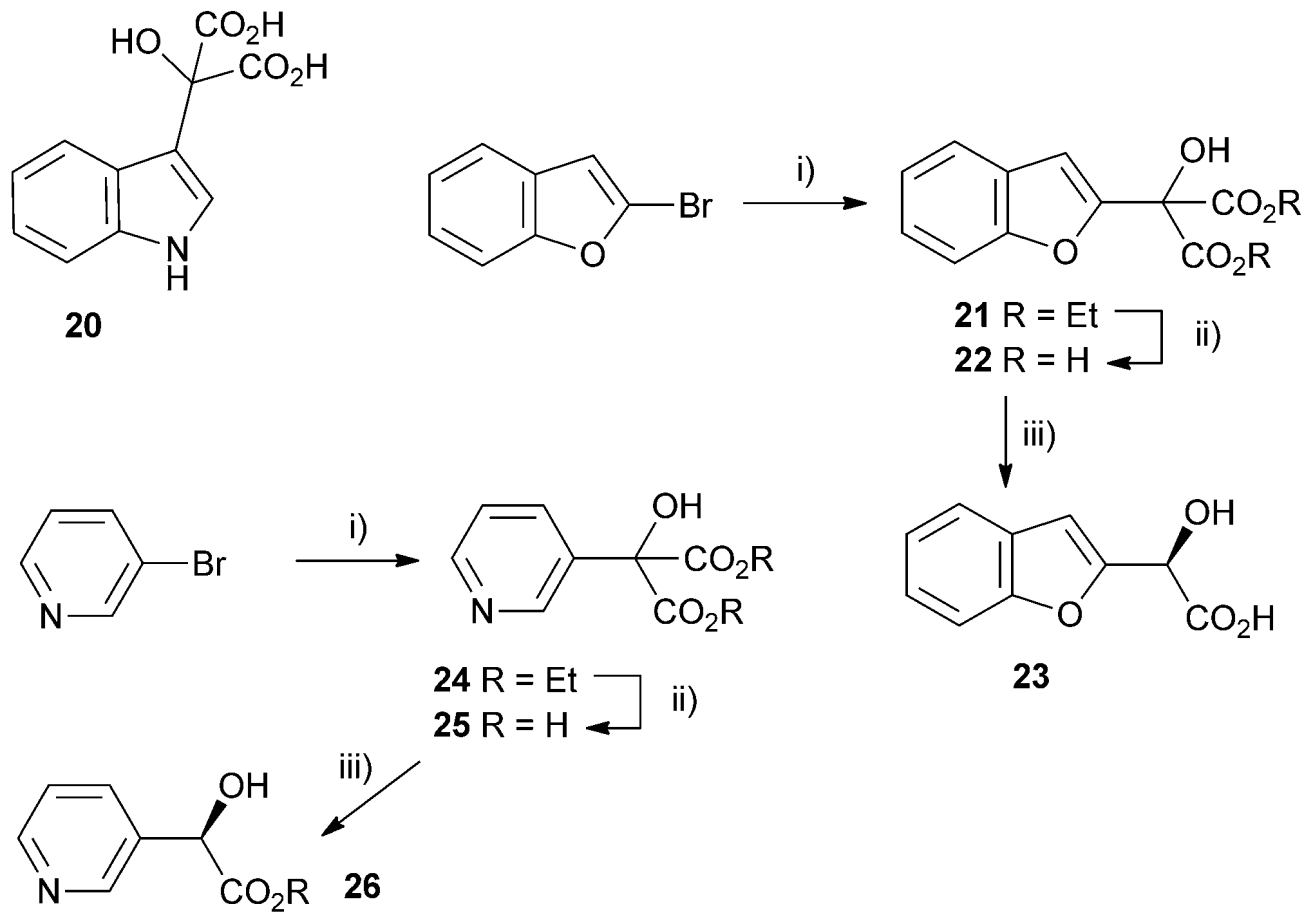
Other heterocyclic α-hydroxy malonate AMDase substrates: i) *n*BuLi, Et_2_O, −78 °C, then diethylketomalonate 6; ii) NaOH (2.2 equiv), dichloromethane/methanol 2 h, RT; iii) malonate substrate (500 mm), AMDase (0.001 mm), DTT (0.05 mm) TRIS buffer (10 mm, pH 7.0), 6 h.

Other heterocyclic α-hydroxy malonic acids were prepared and assayed with wild-type AMDase for decarboxylase activity. Firstly α-(1*H*-indol-3-yl)-α-hydroxy malonic acid **20** was prepared in a manner similar to that of **8 a**, by reaction between the indole and diethyl ketomalonate **6** followed by hydrolysis (Scheme 3). However, **20** was shown not to be a substrate for AMDase, which was not entirely surprising given that substitution of indole in the 3-position is somewhat analogous to substitution of naphthalene in the 1-position, and it has been shown that disubstituted malonic acids with a 1-naphthyl substituent are not substrates for AMDase.[26] Modelling of the active site of AMDases with substrate **20** clearly shows that this is due to steric factors.

Also, α-(benzo[b]furan-2-yl)-α-hydroxy malonic acid **22** was prepared to compare with **8 a**, with the expectation being that extending the aromatic system from a furyl ring to a benzofuryl system would increase delocalisation of negative charge, resulting from decarboxylation, consequently resulting in a greater rate of reaction. The substrate was prepared by treating 2-bromobenzofuran with *n*-butyllithium and then adding diethyl ketomalonate **6** to give α-(benzo[b]furan-2-yl)-α-hydroxy diethyl malonate **21**, which was subsequently treated with sodium hydroxide in methanol/dichloromethane to give the sodium salt of malonate **22** (Scheme 3).

Kinetic parameters obtained with wild-type AMDase confirmed that **22** is decarboxylated four times faster than its furanyl analogue **8 a**, with a *K*_m_ value roughly similar to that of **8 a**. However, contrary to **8 a**–**e**, the M159V and P14V/P15G mutations resulted in a lower enzymatic activity with *k*_cat_ values being 66 and 42 % that of the wild type, respectively. *K*_m_ values were roughly the same for the wild-type and P14V/P15G variant, but improved by a factor of two with the M159V variant.

Similarly, α-hydroxy-α-(pyridin-3-yl) malonic acid **25** was prepared by lithiation of 3-bromopyridine, followed by the addition to diethyl ketomalonate **6** to give the diester **24**, followed by saponification with sodium hydroxide in methanol/dichloromethane to afford the sodium salt of **25** (Scheme 3). Malonate **25** was shown to be an excellent substrate with the wild-type AMDase giving **26** with a *k*_cat_ value approximately 2.2 times that of α-phenyl-α-hydroxy malonic acid (**1**, L=Ph and S=OH) and a *K*_m_ value five times lower. However, kinetic parameters obtained for **25** with the two mutant AMDases were again considerably worse than those obtained with the wild-type enzyme, with *k*_cat_ values of approximately 34 and 19 % of that obtained with the wild-type enzyme for the M159V and P14V/P15G mutants, respectively.

**Scheme 4:**
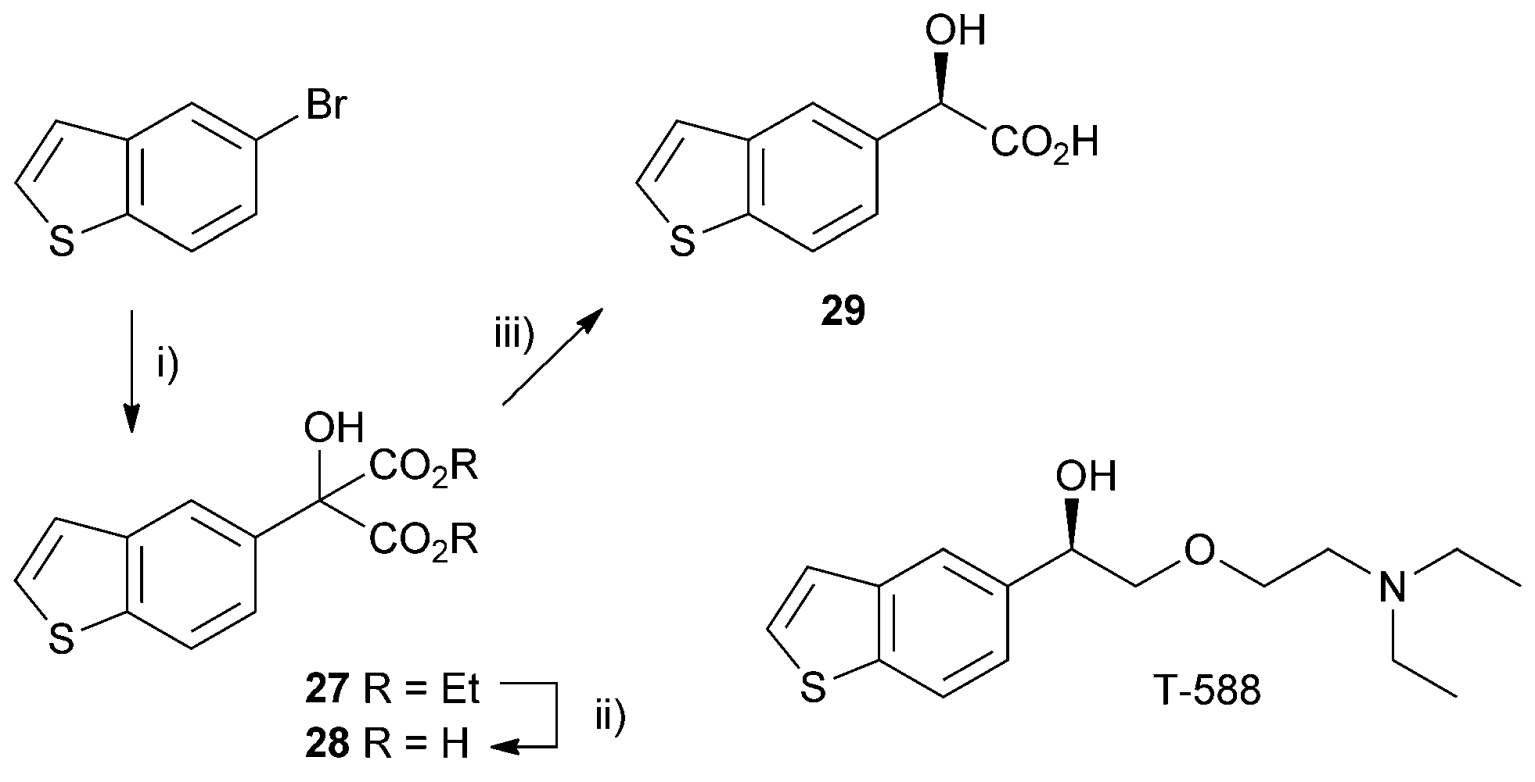
Chemoenzymatic synthesis of the benzothiophenyl precursor 29 required for the synthesis of cognitive enhancer T-588. i) MeI, 1,2-dibromoethane, Mg, Et_2_O, reflux 2 h, then −78 °C, diethylketomalonate 6; ii) NaOH (2.2 equiv), dichloromethane/methanol 2 h, RT; iii) malonate substrate (10 mm), AMDase (0.001 mm), DTT (0.05 mm) TRIS buffer (10 mm, pH 7.0), 6 h.

Finally, α-(benzo[b]thiophen-5-yl)-α-hydroxymalonic acid **28** was chosen as a potential AMDase substrate, because the decarboxylation product **29** has been shown to be a useful intermediate in the synthesis of cognitive enhancer T-588.[21] The substrate, malonate **28**, was derived from the corresponding diester **27**, which itself was synthesised from ketomalonate and the Grignard reagent prepared with 5-bromobenzothiophene **27** (Scheme 4). Kinetic parameters obtained with the wild-type enzyme show an increased rate of catalysis compared to that of α-phenyl-α-hydroxy malonic acid (**1**, L=Ph & S=OH), as would be expected given that **28** has a larger π-system through which to delocalise the negative charge that develops upon decarboxylation. M159V and P14V/P15G mutants again showed much lower rates of catalysis, although *K*_m_ values were much lower than that for α-phenyl-α-hydroxy malonic acid (**1**, L=Ph and S=OH).

## Conclusions

We have described an efficient enzymatic synthesis of homochiral heteroaryl α-hydroxy acetic acids. Reaction rates of furanyl malonates were on the whole higher with the AMDase mutant M159V, whilst the wild-type AMDase gave the highest rates with the other heteroaromatic substrates. The AMDase mutants M159V and P14V/P15G both showed a trend towards lower *K*_m_ values, with the entire set of malonate substrates tested, compared with the *K*_m_ values observed with the wild-type AMDase. Overall, our approach offers a simple, three-step production of α-heterocyclic-α-hydroxy acetic acids in high yields and *ee* from simple heterocyclic precursors and readily available diethyl ketomalonate **6**.

Asymmetric synthesis methodologies have been used to generate enantiopure α-hydroxy acetic acids. For example, a range of α-hydroxy acetic acids, including α-(furan-2-yl)-α-hydroxy acetic acid **9 a**, can be prepared by the diastereoselective reduction of chiral α-keto orthoesters derived from tartaric acid.[27] However, this synthesis requires eight synthetic steps with a modest overall yield for preparation of **9 a**, whereas using the method we have outlined above it is possible to produce **9 a** in only three steps, with a greater overall yield, without the need for as many deleterious solvents and conditions, and with a high *ee*.

Enantioselective Friedel–Crafts reactions between aryl compounds and glyoxylates have been developed that utilise chiral Cu and Ti catalysts,[20, 28–30] and enantioselective arylation of glyoxylates using aryl silanes and chiral Pd catalysts have also been developed.[31] However, in both of these approaches the chiral catalysts are expensive and/or utilise toxic transition metal species. Also, some of the transition-metal catalysts used result in α-hydroxy acetic acids with only modest *ee* relative to our enzymatic method. Asymmetric hydrogenation of aryl α-keto acids using chiral Ru catalysts has also been employed to generate homochiral α-hydroxy carboxylic acids,[21, 32, 33] but Ru compounds are generally considered to be toxic.[34–36] An enzymatic approach offers considerable advantages in this respect. Finally, non-enzymatic enantioselective decarboxylative protonation (EDP) methods have also been developed for decarboxylation of prochiral malonates to generate homochiral acids,[37] with methods utilising both transition-metal catalysis[38] and organocatalysis.[39, 40] However, such methods often require expensive catalysts, complex organic bases, extreme conditions or are largely inefficient and result in low enantioselectivity.[37, 40, 41]

A number of biocatalytic methods have been developed for the production of enantiopure α-hydroxy carboxylic acids, including kinetic resolutions utilising lipase or esterase enzymes.[42, 43] Similarly, nitrilases (or nitrile hydratases/amide hydrolases) can be used to resolve racemic nitriles resulting in α-hydroxy acids.[44–46] However, all of these kinetic resolution approaches are limited to a maximum of 50 % yield of the desired enantiomer. To overcome the limitation in the yields obtained from kinetic resolutions, dynamic kinetic resolution (DKR) methodologies have been developed. For instance, deracemisation of α-hydroxycarboxylic acids has been achieved using a racemase in combination with a lipase.[47, 48] In this case, the lipase selectively acylates one enantiomer of the α-hydroxycarboxylic acid precursor, whilst the racemase racemises the other enantiomer. However, this approach utilises two enzymes that require different solvent conditions, given that the racemase requires an aqueous buffer and the lipase requires a non-aqueous media when used in the synthetic direction. Consequently, technical issues associated with utilising a two-phase system, including the solubility of substrates and products, and the compatibility of the enzymes with the different solvent mixtures become significant. DKR methods have also been used to generate homochiral cyanohydrins.[49, 50] However, the harsh conditions that would be required to hydrolyse cyanohydrins to generate α-hydroxy carboxylic acids could lead to some racemisation of the resultant products.[51] Enantiopure α-hydroxy carboxylic acids can also be prepared from α-keto acids by using dehydrogenases,[52, 53] but this requires co-factor recycling, which can lead to both added complication and additional expense.

In summary, the chemoenzymatic method we have described for the synthesis of enantioenriched α-hydroxy carboxylic acids is a valuable addition to, and in some cases offers advantages over, the existing methodology that has been used to prepare these valuable chiral building blocks.

## Experimental Section

### General

Full experimental details can be found in the Supporting Information.

## Supporting Information

As a service to our authors and readers, this journal provides supporting information supplied by the authors. Such materials are peer reviewed and may be re-organized for online delivery, but are not copy-edited or typeset. Technical support issues arising from supporting information (other than missing files) should be addressed to the authors.

miscellaneous_information
