# Supplementary material for: Enzymatic Enantioselective Decarboxylative Protonation of Heteroaryl Malonates
Source: Chemistry. 2015 Mar 12;21(17):6557–63. doi: 10.1002/chem.201406014 (PMC4517146; doi:10.1002/chem.201406014)
Supplement: Supplementary file 1 — miscellaneous_information [file chem0021-6557-sd1.pdf]

CHEMISTRY
A European Journal

Supporting Information

Enzymatic Enantioselective Decarboxylative Protonation of Heteroaryl Malonates

Ross Lewin,[a] Mark Goodall,[a] Mark L.Thompson,[a] James Leigh,[a] Michael Breuer,[b] Kai Baldenius,[b] and Jason Micklefield*[a]

chem_201406014_sm_miscellaneous_information.pdf

SUPPORTING INFORMATION

Contents

S2: Additional experimental methods for the synthesis of malonate substrates.

S13: Determination of the absolute configuration of selected α-hydroxy carboxylic acids using Mosher esters, which is consistent with the previously determined stereochemical course of AMDase catalysed reactions.[1,2]

S17: Figure S1 illustrates the synthesis, separation and analysis of Mosher esters of selected α-hydroxy carboxylic acids.

S17: Table S1 shows example of NMR data of Mosher's esters derived from α-hydroxy-α-(thiophen-2-yl) acetic acid **19**.

S18: CD spectra of α-hydroxy-α-heteroaryl acetic acids

Experimental

Synthesis α-(Furan-2-yl)-α-hydroxy diethyl malonates (7a-e): The general procedure used to synthesise the α-(furan-2-yl)-α-hydroxy diethyl malonates **7a-e** is illustrated by the synthesis of α-(furan-2-yl)-α-hydroxy diethyl malonate **7a**.[24]

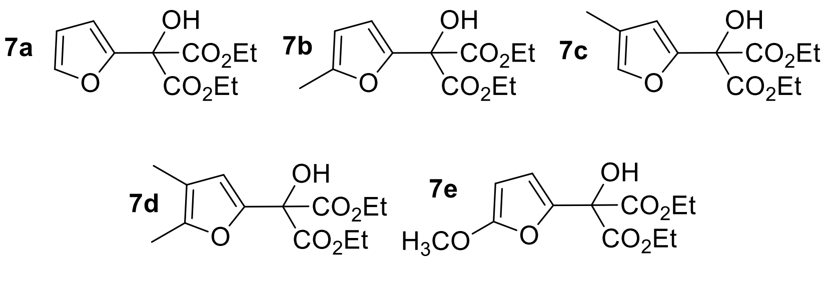

Accordingly, diethyl ketomalonate **6** (2.0 g, 1.75 mL, 0.011 mol) was cooled to 0 °C prior to the drop wise addition of furan **5a** (neat, 0.78 g, 0.84 mL, 0.011 mol). The solution was then stirred overnight at room temperature. The product was purified by flash chromatography using a 7:1 mixture of hexane and ethyl acetate as the eluent the product **7a** (1.3 g, 45%) as a yellow oil.

α-(Furan-2-yl)-α-hydroxy diethyl malonate (**7a**):[24] ^{1}H NMR (400 MHz, $CDCl_3$) δ = 1.32 (t, J = 7.1 Hz, 6H, CH_3), 4.35 (m, 4H, CH_2), 6.39 (dd, J = 3.4, 1.8 Hz, 1H; Ar-H), 6.62 (dd, J = 3.4, 0.8 Hz, 1H; Ar-H), 7.44 (dd, J = 1.8, 0.8 Hz, 1H; Ar-H); ^{13}C NMR (100 MHz, $CDCl_3$,) δ = 13.94 (CH_3) 63.26 (CH_2), 76.51 (COH), 109.80 (Ar-C), 110.40 (Ar-C), 143.31 (Ar-C), 148.70 (Ar-C), 168.01 (C=O); HRMS (ESI) *m/z*: Calculated for $C_{11}H_{14}O_6$ $[M + Na]^+$, 265.0683; Found, 265.0686.

α-Hydroxy-α-(5-methylfuran-2-yl) diethyl malonate (**7b**),[S1] was prepared according to the above procedure with **6** (2.0 g 0.011 mol) and 2-methylfuran (0.902 g, 0.011 mol) to give the product **7b** (1.098 g, 39%). ^{1}H NMR ($CDCl_3$, 400 MHz) δ 1.32 (t, J = 7.1 Hz, 6H; CH_3), 2.29 (3H, s; Ar-CH_3), 4.35 (q, J = 7.1 Hz, 4H; CH_2), 5.95 (m, 1H; Ar-H), 6.48 (d, J = 3.3 Hz, 1H; Ar-H); ^{13}C NMR ($CDCl_3$, 75 MHz) δ 13.57 (Ar-CH_3), 13.94 (CH_3), 63.13 (CH_2), 76.49 (COH), 106.45 (Ar-H), 110.77 (Ar-H), 146.79 (Ar-C), 153.22 (Ar-C), 168.14 (C=O); HRMS (ESI) *m/z*: Calculated for $C_{12}H_{16}O_6$ $[M + Na]^+$, 279.0840; Found: 279.0847.

α-Hydroxy-α-(4-methylfuran-2-yl) diethyl malonate (**7c**), was prepared according to the above procedure with **6** (2.0 g 0.011 mol) and 3-methylfuran (0.902 g, 0.011 mol) to give the product **7c** (1.126 g, 40%). ^{1}H NMR ($CDCl_3$, 300 MHz) δ 1.34 (t, J = 6.0 Hz, 6H; CH_3),

2.06 (s, 3H; Ar-CH_3), 4.38 (m, 4H; CH_2), 6.26 (d, J = 1.6 Hz, 1H; Ar-H) 7.31 (d, J = 1.6 Hz, 1H; Ar-H). ^{13}C NMR ($CDCl_3$, 75 MHz); δ 10.29 (Ar-CH_3), 13.98 (CH_3), 63.25 (CH_2), 77.18 (COH), 114.29 (Ar-C), 119.37 (Ar-C), 141.49 (Ar-C), 143.76 (Ar-C), 168.48 (C=O); HRMS (ESI) *m/z*: Calculated for $C_{12}H_{16}O_6$ [M + Na]$^+$: 279.0840; Found: 279.0840.

α-(4,5-Dimethylfuran-2-yl)-α-hydroxy diethyl malonate **(7d)**, was prepared according to the above procedure with **6** (2.0 g 0.011 mol) and 2,3-dimethylfuran (1.056 g, 0.011 mol) to give the product **7d** (1.129 g, 38%). ^{1}H NMR ($CDCl_3$, 300 MHz) δ 1.24 (t, J = 7.1 Hz, 6H; CH_3), 1.85 (s, 3H; Ar-CH_3), 2.12 (s, 3H; Ar-CH_3), 4.27 (m, 4H; CH_2), 6.30 (s, 1H; Ar-H). ^{13}C NMR ($CDCl_3$, 75 MHz); δ 9.83 (Ar-CH_3), 11.40 (Ar-CH_3), 13.96 (CH_3), 63.13 (CH_2), 76.50 (COH), 113.17 (Ar-CH), 113.43 (Ar-C), 145.51 (Ar-C), 148.62 (Ar-C), 168.20 (C=O); HRMS (ESI) *m/z*: Calculated for $C_{13}H_{18}O_6$ [M + Na]$^+$: 293.0996 Found: 293.1006.

α-Hydroxy-α-(5-methoxyfuran-2-yl) diethyl malonate **(7e),** was prepared according to the above procedure with **6** (2.0 g 0.011 mol) and 2-methoxyfuran (1.078 g, 0.011 mol) to give the product **7d** (0.748 g, 25%). ^{1}H NMR ($CDCl_3$, 300 MHz) δ 1.33 (t, J = 7.0 Hz, 6H; CH_3), 3.84 (s, 3H; OCH_3), 4.34 (m, 4H; CH_2), 5.15 (d, J = 3.4 Hz, 1H; Ar-H), 6.50 (d, J = 3.4 Hz, 1H; Ar-H). ^{13}C NMR ($CDCl_3$, 75 MHz) δ 13.94 (CH_3), 57.79 (OCH_3), 63.15 (CH_2), 76.27 (COH), 80.46 (Ar-C), 111.68 (Ar-C), 138.40 (Ar-C), 161.89 (Ar-C), 168.02 (C=O). LR-MS (ES, m/z): Calculated for $C_{12}H_{18}O_7$ [M + H]$^+$: 273.1 Found: 273.0.

α-thiophen-2-yl-α-hydroxy, α-(benzofuran-2-yl)-α-hydroxy and α-Hydroxy-α-(pyridin-3-yl) diethyl malonates (17, 21 and 24): These compounds were prepared *via* the lithiation of 3-bromopyridine, 2-bromobenzofuran and thiophene, respectively. The general procedure used to synthesise these compounds will be illustrated by the synthesis of α-(benzofuran-2-yl)-α-hydroxy diethyl malonate **21.**

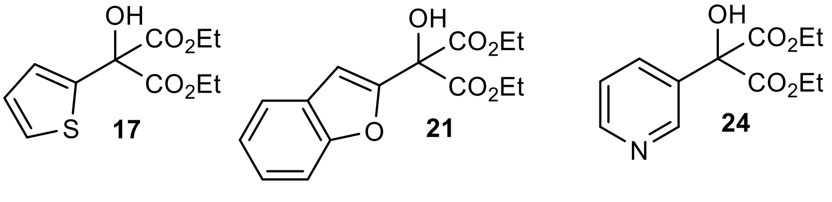

2-Bromobenzofuran (0.275 g, 1.36 mmol) and anhydrous diethyl ether (7.0 mL) were added to a flame-dried flask, under nitrogen, cooled to –78 °C before the dropwise addition of *n*BuLi (1.6 M in hexanes, 0.851 mL, 1.36 mmol). The resulting mixture was stirred for 20 minutes at –78 °C and then a solution of diethyl ketomalonate **6** (0.287 g, 1.64 mmol) in

anhydrous diethyl ether (3.0 mL) was added dropwise at –78 °C. The reaction mixture was allowed to warm to room temperature, then stirred overnight, before being quenched with saturated ammonium chloride solution (5.0 mL). The mixture was then extracted with ethyl acetate (3 x 30 mL) and the combined organic extracts were then dried over anhydrous $MgSO_4$, before being evaporated under reduced pressure, to give the crude product as an oil. The product was purified by flash chromatography using a 10:1 mixture of hexane and ethyl acetate as the eluent to give **21** (0.15 g, 38%) as a pale yellow oil.

α-(Benzofuran-2-yl)-α-hydroxy diethyl malonate **(21)**, ^{1}H NMR ($CDCl_3$, 400 MHz) δ 1.24 (t, 6H, *J* = 7.1 Hz; CH_3), 4.34 – 4.23 (m, 4H; CH_2), 6.95 (s, 1H; C3-H), 7.16 (m, 1H, C5-H), 7.23 (m, 1H, C6-H), 7.41 (d, 1H, *J* = 8.3 Hz; C7-H), 7.51 (d, 1H, J = 7.7 Hz; C4-H). ^{13}C NMR ($CDCl_3$, 100 MHz) δ 13.97 (CH_3), 63.51 (CH_2), 106.61 (C3), 111.53 (C7), 121.60 (C4), 123.07 (C5), 124.98 (C6), 127.52 (C8), 151.18 (C2), 155.11 (C9), 167.75 (COOEt). IR (neat) 3467, 2984, 1736, 1453, 1215 cm^{-1}. HR-MS (ES, m/z): Calculated for $C_{15}H_{16}O_6$ [M - H]$^-$: 291.0874 Found: 291.0870.

α-Thiophen-2-yl-α-hydroxy diethyl malonate **(17)**, was prepared has described above with *n*-butyllithium (1.6M, 3.71 mL, 5.94 mmol), thiophene (0.500g, 5.94mmol) and (diethyl)ketomalonate (1.242g, 7.13mmol) in THF (30 mL). Purification by flash chromatography (hexane:Ethyl Acetate; 5:1) gave **17** as a yellow oil (0.430g, 28%). ^{1}H NMR ($CDCl_3$, 400 MHz) δ 1.23 (t, 6H, *J* = 7.1 Hz; CH_3), 4.31-4.17 (m, 4H; CH_2), 4.50 (s, 1H; OH), 6.94 (dd, *J* = 5.0, 3.7 Hz, 1H; Ar-H), 7.26-7.22 (m, 2H; Ar-H). ^{13}C NMR ($CDCl_3$, 100 MHz) 13.92 (CH_3), 63.82 (CH_2), 78.35 (COH), 126.21 (Ar-C), 126.83 (2x Ar-C), 139.13 (Ar-C) 168.88 (*C*OOEt). IR (neat) 3469, 2981, 1730, 1205 cm^{-1}. HR-MS (ES, m/z): Calculated for $C_{11}H_{14}O_5S$ [M + H]$^+$: 259.0635 Found: 259.0635.

α-Hydroxy-α-(pyridin-3-yl) diethyl malonate **(24)**, was prepared has described above with *n*-butyllithium (1.6M, 2.60 mL, 4.13 mmol), 3-bromopyridine (0.652 g, 4.13 mmol) and (diethyl)ketomalonate (0.717 g, 4.1 mmol) in diethylether (13mL). Purification by flash chromatography, (eluted with 2:1 hexane:ethyl aceteate) gave **24** (0.314 g, 30%). ^{1}H NMR ($CDCl_3$, 400 MHz) δ 1.19 (t, 6H, *J* = 1.1 Hz; CH_3), 4.24 (m, 4H; CH_2), 6.28 – 5.64 (s, 1H; OH), 7.24 (dd, 1H, *J* = 8.2, 4.6 Hz; C5-H), 7.98 (ddd, 1H, *J* = 8.2, 2.1, 1.6 Hz; C4-H), 8.48 (dd 1H, *J* = 4.6, 1.6 Hz; C6-H), 8.84 (d, 1H, *J* = 2.1 Hz; C2-H). ^{13}C NMR ($CDCl_3$, 100 MHz) δ 13.87 (CH_3), 63.06 (CH_2), 78.88 (COH), 122.79 (C5), 132.31 (C3), 134.91 (C4), 148.19 (C2), 149.11 (C6), 169.26 (COOEt). IR (neat) 3467, 2984, 1735, 1367, 1216 cm^{-1}. HR-MS (ES, m/z): Calculated for $C_{12}H_{15}NO_5$ [M + H]$^+$: 254.1023 Found: 254.1028.

α-(Benzo[b]thiophen-5-yl)-α-hydroxy diethyl malonate (27):

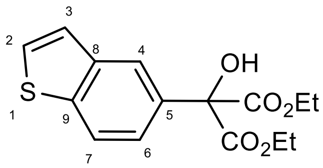

27

A solution was prepared comprising of 5-bromobenzothiophene (0.419 g, 1.9 mmol), methyl iodide (1.1 g, 7.8 mmol), 1,2-dibromoethane (0.1 g, 0.5 mmol) and diethyl ether (10.0 mL). 10% of this solution was then added to a flask containing diethyl ether (2 mL), pre-heated magnesium turnings (0.427 g, 17 mmol) and a few iodine crystals. This mixture was heated and allowed to reflux until the iodine colour faded, after which the rest of the 5-bromobenzothiophene solution was added in 10% portions. When the addition was complete, the mixture was heated under reflux for 2 hours, after which time the solution was cooled to – 78 °C. Diethyl ketomalonate **6** (0.342 g, 1.966 mmol) was then added dropwise to the solution, which immediately turned orange in colour. The solution was allowed to warm to room temperature and then stirred overnight, before being quenched with a saturated ammonium chloride solution (10 mL). The resulting mixture was extracted with diethyl ether (3 x 30 mL) and the combined organic layers were then washed with brine (10 mL), dried over anhydrous magnesium sulphate, and solvent subsequently removed under reduced pressure. The crude product was purified by flash chromatography using a 5:1 mixture of hexane and ethyl acetate as the eluent to give malonate **27** (0.12 g, 20%) as a yellow oil. ^{1}H NMR (400 MHz, $CDCl_3$) δ 1.22 (t, *J* = 7.1 Hz, 6H; CH_3), 4.24 (m, 4H; CH_2), 4.40 (s, 1H; OH), 7.28 (dd, *J* = 5.4, 0.4 Hz, 1H; C2-H), 7.38 (dd, *J* = 5.4, 1.6 Hz, 1H; C3-H), 7.54 (dd, *J* = 8.6, 1.6 Hz, 1H, C6-H), 7.80 (d, *J* = 8.6 Hz, 1H; C7-H), 8.07 (d, *J* = 1.6 Hz, 1H; C4-H); ^{13}C NMR (101 MHz, $CDCl_3$) δ 13.98 (CH_3), 63.10 (CH_2), 80.04 (COH), 121.67 (C3), 121.98 (C7), 123.07 (C2), 124.24 (C4), 127.01 (C6), 132.22 (C8), 139.33 (C5), 139.98 (C9), 170.03 (COOEt) IR (neat) 3469, 2981, 1729, 1204, 1252 cm^{-1}. HR-MS (ES, m/z): Calculated for $C_{15}H_{16}O_5S$ $[M+H]^+$ 309.0791 Found: 309.0802.

α-Furyl-α-hydroxy malonic acids (8a-e). The general procedure used to synthesise the α-furyl-α-hydroxy malonic acids **8a-e** *via* hydrolysis of their respective diethyl malonates will be illustrated by the synthesis of α-(furan-2-yl)-α-hydroxymalonic acid **8a**.

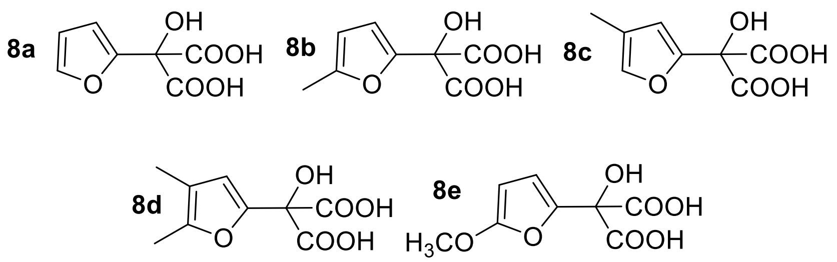

A solution of NaOH (0.344 g, 8.61 mmol) in H_2O (10 mL) was added to 2-(furan-2-yl)-2-hydroxy diethyl malonate **7a** (1.0 g, 4.10 mmol) in EtOH (5 mL). The resulting mixture was heated under reflux for three hours. After this, the solution was cooled to 0 °C and then adjusted to pH 7.0 using dilute aqueous HCl (10% v/v). The mixture was washed with diethyl ether (3 x 30 mL), and then the aqueous extracts were frozen and lyophilised to obtain the malonic acid **8a** (0.724 g, 95%) as a pale yellow solid.

α-(Furan-2-yl)-α-hydroxymalonic acid **(8a)**: ^{1}H NMR (D_2O, 400 MHz) δ 6.37 (m, 2H; Ar-H), 7.43 (s, 1H; Ar-H); ^{13}C NMR (D_2O, 100 MHz) δ 79.79 (COH), 108.00 (Ar-H), 110.41 (Ar-H), 142.54 (Ar-H), 154.11 (Ar-C), 175.36 (COOH); LRMS (ESI) *m/z*: Calculated for $C_7H_6O_6$ $[M + H]^+$: 186.0 Found: 186.0.

α-Hydroxy-α-(5-methylfuran-2-yl)malonic acid **(8b)**, was prepared according to the above procedure with NaOH (0.378 g, 8.19 mmol) and **7b** (1.0 g 3.90 mmol) to give the product **8b** (0.741 g, 95%). ^{1}H NMR (D_2O, 400 MHz) δ 2.14 (s, 3H; Ar-CH_3), 5.88 (m, 1H; Ar-H), 6.14 (d, J = 3.1 Hz, 1H; Ar-H); ^{13}C NMR (D_2O, 100 MHz) δ 12.65 (Ar-CH_3), 79.73 (COH), 105.83 (Ar-C), 108.91 (Ar-C), 152.18 (Ar-C), 152.39 (Ar-C), 175.39 (COOH); LRMS (ESI) *m/z*: Calculated for $C_8H_8O_6$ $[M – COOH – H]^-$: 157.1 Found: 157.0.

α-Hydroxy-α-(4-methylfuran-2-yl)malonic Acid **(8c)**, was prepared according to the above procedure with NaOH (0.378 g, 8.19 mmol) and **7c** (1.0 g 3.90 mmol) to give the product **8c** (0.764 g, 98%). ^{1}H NMR (D_2O, 400 MHz) δ 1.84 (s, 3H; Ar-CH_3), 6.18 (d, J = 1.5 Hz, 1H; Ar-H), 7.22 (d, J = 1.5 Hz, 1H; Ar-H); ^{13}C NMR (D_2O, 100 MHz) δ 9.63 (Ar-CH_3), 79.13 (COH), 113.99 (Ar-C), 117.43 (Ar-C), 140.59 (Ar-C), 148.65 (Ar-C), 175.23 (COOH); LRMS (ESI) *m/z*: Calculated for $C_8H_8O_6$ $[M + H]^+$: 201.0 Found: 201.0.

α-(4,5-Dimethylfuran-2-yl)-α-hydroxymalonic Acid **(8d)**, was prepared according to the above procedure with NaOH (0.310 g, 7.77 mmol) and **7d** (1.0 g 3.70 mmol) to give the product **8c** (0.744 g, 94%). ^{1}H NMR (D_2O, 400 MHz) δ 1.75 (s, 3H; Ar-CH_3), 2.02 (s, 3H; Ar-CH_3), 6.02 (s, 1H, Ar-H); ^{13}C NMR (D_2O, 75 MHz) δ 8.85 (Ar-CH_3), 10.38 (Ar-CH_3),

79.65 (COH), 111.27 (Ar-C), 114.37 (Ar-C), 147.22 (Ar-C), 150.92 (Ar-C), 175.40 (COOH); LRMS (ESI) *m/z*: Calculated for $C_9H_{10}O_6$ $[M - H]^-$: 213.0 Found: 213.0.

α-Hydroxy-α-(5-methoxyfuran-2-yl)malonic Acid **(8e),** was prepared according to the above procedure with NaOH (0.310 g, 7.77 mmol) and **7e** (1.0 g 3.70 mmol) to give the product **8e** (0.759 g, 95%). ^1H NMR (D_2O, 400 MHz) δ 3.73 (s, 3H; OCH_3), 5.12 (d, J = 3.2 Hz, 1H; Ar-H), 6.14 (d, J = 3.2 Hz, 1H; Ar-H). ^{13}C NMR (D_2O, 100 MHz) δ 58.86 (OCH_3), 79.69 (COH), 79.93 (Ar-C), 109.71 (Ar-C), 143.89 (Ar-C), 160.79 (Ar-C), 175.13 (COOH); LRMS (ESI) *m/z*: Calculated for $C_8H_8O_7$ $[M + H]^+$: 216.1 Found: 216.0.

α-Acetoxy-α-(furan-2-yl) diethyl malonate (10):[24]

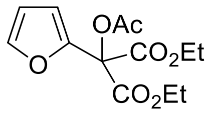

10

Acetic anhydride (0.42 g, 0.392 mL, 4.2 mmol), triethylamine (0.13 g, 0.175 mL, 1.26 mmol) and DMAP (0.028 g, 0.227 mmol) were slowly added to a stirred solution of α-(furan-2-yl)-α-hydroxy diethyl malonate **7a** (0.10 g, 0.42 mmol) in dry DCM (10 mL). The reaction mixture was then stirred for 48 hours at room temperature. The mixture was then diluted with H_2O (40 mL) and solid $NaHCO_3$ was added, under vigorous stirring, until no further CO_2 was generated. The resulting solution was then extracted with diethyl ether (3 x 30 mL) and the combined organic extracts were dried over anhydrous $MgSO_4$ and then evaporated under reduced pressure. The crude product was purified by flash chromatography using a 7:1 mixture of hexane and ethyl acetate as the eluent to give **10** (1.13 g, 95%) as an oil. ^1H NMR ($CDCl_3$, 400 MHz) δ 1.31 (t, J = 6.1 Hz, 6H; CH_3), 2.22 (s, 3H; CH_3), 4.35 (q, J = 6.1 Hz, 4H; CH_2), 6.43 (d, J = 3.1 Hz, 1H; Ar-H), 6.71 (dd, J = 1.5, 3.1 Hz, 1H; Ar-H), 6.71 (d, J = 1.5 Hz, 1H; Ar-H); ^{13}C NMR ($CDCl_3$, 100 MHz) δ 13.89 (CH_3), 20.80 (CH_3), 63.01 (CH_2), 85.04 (COAc), 110.82 (Ar-C), 111.82 (Ar-C), 143.56 (Ar-C), 150.51 (Ar-C), 164.05 (COOH), 177.06 (C=O); HRMS (ESI) *m/z*: Calculated for $C_{13}H_{16}O_7$ $[M + Na]^+$: 307.0789 Found: 307.0792.[24]

α-(Furan-2-yl) malonic acid (12)[24]:

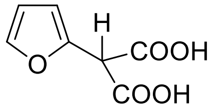

12

A mixture of sodium metal (1.83 g, 10.44 mmol) and α-(dimethylamino)naphthalene (11.38 g, 10.92 mL, 10.21 mmol) in DMPU (20 mL) was stirred for 15 hours at room temperature. After this, a solution of α-acetoxy-α-(furan-2-yl) diethyl malonate **10** (0.10 g, 0.35 mmol) in dry toluene (10 mL) was added. The reaction mixture was stirred for a further 60 minutes before being filtered to remove any remaining sodium metal. The filtrate was diluted with aqueous HCl (30 mL 10% v/v) before being extracted with diethyl ether (3 x 30 mL). The combined organic extracts were dried over $MgSO_4$ and then evaporated to yield the crude α-(furan-2-yl) diethyl malonate **11**. The intermediate **11** was subsequently dissolved in EtOH (5 mL) and KOH (0.041 g, 0.735 mmol) in H_2O (10 mL) was added, with the mixture then being heated under reflux for three hours. The mixture was then cooled to 0 °C and adjusted to pH 7 using dilute aqueous HCl (10% v/v), washed with diethyl ether (3 x 30 mL), and the aqueous layer was extracted, frozen and lyophilised to obtain the product **12** (0.047 g, 79%) as a pale yellow solid. ^{1}H NMR ($CDCl_3$, 400 MHz) δ 1.95 (s, 1H; CH), 6.29 (dd, J = 1.5, 3.1 Hz, 1H; Ar-H), 6.46 (d, J = 3.1 Hz, 1H; Ar-H), 6.29 (d, J = 1.5 Hz, 1H; Ar-H); ^{13}C NMR ($CDCl_3$, 75 MHz) δ 71.07 ($CH(CO_2H)_2$), 91.49 (Ar-C), 130.53 (Ar-C), 131.81 (Ar-H), 156.66 (Ar-C), 178.92 (COOH); LRMS (ESI) *m/z*: Calculated for $C_7H_6O_5$ $[M + H]^+$: 171.0 Found: 171.0.[24]

Heteroaromatic α-hydroxy malonic acids (18, 22, 25 and 28): The general procedure for the synthesis of the other α-hydroxy malonic acids *via* hydrolysis of their respective diethyl malonates will be illustrated by the synthesis of α-(benzofuran-2-yl)-α-hydroxy diethyl malonate **22**.

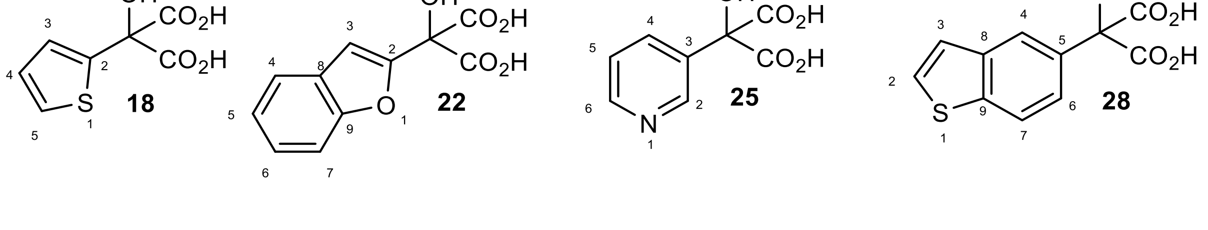

α-(benzofuran-2-yl)-α-hydroxy diethyl malonate **21** (0.100 g, 0.342 mmol) in dichloromethane (4.5 mL) was added to a solution of sodium hydroxide (0.030 g, 0.753 mmol) in methanol (0.5 mL) in a centrifuge tube. The mixture was allowed to stand for 3 hours, after which a precipitate had formed. The tube was centrifuged (5000 RPM, 3 minutes), the supernatant was poured off, and the solid pellet that had formed was washed with ethyl acetate (4 mL) and diethyl ether (8 mL). The remaining solvent was removed *in vacuo* to yield the disodium salt of α-(benzofuran-2-yl)-α-hydroxy malonic acid **22** (0.085 g, 90%) as a white powder.

α-(Benzofuran-2-yl)-α-hydroxy malonic acid **(22)**: ^{1}H NMR (400 MHz, D_2O) δ 6.73 (s, 1H; C3-H), 7.17 (m, 1H; C5-H), 7.24 (m, 1H; C6-H), 7.42 (d, J = 7.7 Hz, 1H; C7-H), 7.54 (d, J = 6.5 Hz, 1H; C4-H). ^{13}C NMR (101 MHz, D_2O) δ 104.69 (COH), 111.17 (C3), 121.40 (C7), 122.85 (C4), 123.01 (C5), 124.41 (C6), 124.70 (C8), 127.94 (C2), 154.43 (C9), 174.70 (COOH), IR (neat) 3329, 1633, 1317, 1250, 1115 cm^{-1}. HR-MS (ES, m/z): Calculated for $C_{11}H_8O_6$ [M+H]$^+$: 237.0394 Found: 237.0397.

α-(Thiophen-2-yl)-α-hydroxy malonic acid **(18)**, was prepared according to the procedure above with **17** (0.100 g, 0.39 mmol) in DCM (4.5 mL) and NaOH (0.034 g, 0.85 mmol) in methanol (0.5mL), resulting in **18** (0.091g, 95%) as a white solid. ^{1}H NMR (400 MHz, D_2O) δ 6.89 (dd, *J* = 3.5, 5.1 Hz, 1H; C4-H), 7.04 (d, *J* = 3.5 Hz, 1H; Ar-H), 7.23 (d, *J* = 5.1 Hz, 1H; Ar-H). ^{13}C NMR (101 MHz, D_2O) δ 81.58 (COH) 125.11 (Ar-C) 125.41 (Ar-C), 126.51 (Ar-C), 145.20 (Ar-C), 176.47 (COOH). IR (neat) 3377, 1613, 1380, 1320, 1101 cm^{-1}. HR-MS (ES, m/z) Calculated for $C_7H_6O_5S$ [M+H]$^+$: 203.00142 Found: 203.0012

α-(Pyridin-3-yl)-α-hydroxy malonic acid **(25)**, was prepared according to the procedure above with **24** (0.100 g, 0.39mmol) in DCM (4.5mL) and NaOH (0.035 g, 0.87 mmol) in methanol (0.5mL) to give **25** (0.091g, 96%) as a white solid. ^{1}H NMR (400 MHz, D_2O) δ 7.31 (dd, J = 8.0, 4.9 Hz, 1H; C5-H), 7.90 (d, J = 8.0 Hz, 1H; C4-H), 8.31 (d, J = 4.9 Hz, 1H; C6-H), 8.57 (s, 1H; C2-H). ^{13}C NMR (101 MHz, D_2O) δ 82.48 (COH), 123.47 (C5), 136.08 (C3), 137.55 (C4), 137.71 (C2), 147.19 (C6), 176.65 (COOH), IR (neat) 3461, 1631, 1453, 1328, 1180 cm^{-1}. LR-MS (ES, m/z) Calculated for $C_8H_7O_5N$ [M+H]$^+$: 198.0. Found: 198.0.

α-(Benzo[b]thiophen-5-yl)-α-hydroxy malonic acid **(28)**: was prepared according to the procedure above with **27** (0.100 g, 0.32 mmol) in DCM (4.5 mL) and NaOH (0.029 g, 0.73 mmol) in methanol (0.5 mL) to give **28 (**0.086 g, 90%) as a white solid. ^{1}H NMR (400 MHz, D_2O) δ 7.34 (d, *J* = 5.4 Hz, 1H; C3-H), 7.41 (dd, *J* = 8.6, 1.0 Hz, 1H: C6-H), 7.49 (d, *J* = 5.4 Hz, 1H: C2-H), 7.83 (d, *J* = 8.6 Hz, 1H: C7-H) 7.88 (d, *J* = 1.0 Hz, 1H: C4-H) ^{13}C NMR (101

MHz, D_2O) δ 66.52 (COH) 121.79 (C3), 121.94 (C7), 124.02 (C2) 124.28 (C4), 127.16 (C6), 129.48 (C8), 138.27 (C5), 139.18 (C9), 177.62 (COOH), IR (neat) 3313, 1599, 1435, 1359, 1317, 1083 cm^{-1} LR-MS (ES, m/z) Calculated for $C_{11}H_8O_5S$ $[M+Na]^+$ 275.0, Found: 275.0.

Screening malonic acid derivatives as substrates for AMDase: New AMDase substrates were initially identified using the BTB colorimetric assay previously reported.[1,2] In a 96-well plate, 10 μL of the candidate substrate (0.5 M in 25 mM TRIS buffer at pH 7) was added to 185 μL of a BTB-containing buffer solution (0.01% BTB in 10 mM MOPS buffer at pH 7.2). The 96-well plate was then incubated at 37 °C for 15 minutes before the addition of AMDase (*ca.* 1 μM). The absorbance at 620 nm was recorded over the course of 6 hours using a UV-Vis photospectrometer, with an increase in absorbance being indicative of a positive result. In order to verify positive hits from the colorimetric assay, a second assay was performed using ^{1}H NMR. In an Eppendorf tube, 50 μL of the candidate substrate (0.5 M in 25 mM TRIS buffer at pH 7) was added to 925 μL of TRIS buffer (25 mM at pH 7). The Eppendorf tube was then incubated at 37 °C for 15 minutes before the addition of AMDase (*ca.* 10 μM). The solution was incubated overnight at 37 °C before being frozen and lyophilised, with the resultant solids being dissolved in an appropriate deuterated solvent and submitted for ^{1}H NMR analysis.

Preparation of heteroaromatic α-hydroxy carboxylic acids (9a-e, 13, 19, 23, 26, 29) using AMDAse: The substrate (10.0 mg) was dissolved in TRIS buffer (25 mM at pH 7) and then incubated at 37 °C for 15 minutes before the addition of AMDase (*ca.* 10 μM). The reaction was incubated at 37 °C overnight, after which methanol (1 mL) was added to precipitate the enzyme so that it could be removed by centrifugation (13,000 RPM, 1 minute). The supernatant was subsequently purified by reverse-phase HPLC using a Phenomenex C_{18} Gemini (5 μm particle size, 110 Å) column. The solvents used to achieve separation were (A) water containing 0.05% TFA and (B) acetonitrile containing 0.05% TFA with the elution gradient formed as follows: (A):(B) 95:5 from 0 to 5 min; 95:5 to 5:95 from 5 to 15 min; 5:95 from 15 to 20 min; 95:5 from 20 to 25 minutes. The flow rate was 5 mL/min. UV detection was performed between 230 and 260 nm dependent on the compound.

(R)-α-(furan-2-yl)-α-hydroxyacetic acid **(9a)**,[27] was prepared following the general procedure described above with **8a** (10 mg, 0.05 mmol), to give **9a** (7.0 mg, 92%) as a white solid. ^{1}H NMR (D_2O, 400 MHz) δ 5.14 (s, 1H; CH), 6.24 (m, 1H; Ar-H), 6.28 (m, 1H; Ar-H), 7.30 (s, 1H; Ar-H); ^{13}C NMR (D_2O, 100 MHz) δ 66.21 (COH), 109.42 (Ar-C), 110.70 (Ar-C), 143.69 (Ar-C), 150.32 (Ar-C), 174.10 (COOH); LRMS (ESI) *m/z*: Calculated for $C_6H_6O_4$ $[M + H]^+$: 142.1 Found: 143.0; Chiral-HPLC Retention Time: (*R*) enantiomer = 2.900 min. $[\alpha]_D^{25}$ = -59.2° (H_2O).

(R)-α-(5-methylfuran-2-yl)-α-hydroxyacetic acid **(9b)**, was prepared following the general procedure described above with **8b** (10 mg, 0.05 mmol), to give **9b** (6.5 mg, 83%) as a white solid. ^{1}H NMR (D_2O, 400 MHz) δ 2.13 (s, 3H; Ar-CH_3), 5.17 (s, 1H; CH), 5.94 (m, 1H; Ar-H), 6.26 (m, 1H; Ar-H); ^{13}C NMR (D_2O, 100 MHz) δ 12.47 (Ar-CH_3), 66.45 (COH), 106.37 (Ar-C), 110.37 (Ar-C), 148.81 (Ar-CH_3), 153.76 (Ar-C), 174.53 (COOH); LRMS (ESI) *m/z*: Calculated for $C_7H_8O_4$ $[M + H]^+$: 157.1 Found: 157.0; Chiral-HPLC Retention Time: (*R*) enantiomer = 3.424 min. $[\alpha]_D^{25}$ = -46.7° (H_2O)

(R)-α-(4-methylfuran-2-yl)-α-hydroxyacetic acid **(9c)**, was prepared following the general procedure described above with **8c** (10 mg, 0.05 mmol), to give **9c** (7.4 mg, 95%) as a white solid. ^{1}H NMR (D_2O, 400 MHz) δ 1.95 (s, 3H; Ar-CH_3), 5.31 (s, 1H, CH), 6.27 (m, 1H; Ar-H), 7.31 (m, 1H; Ar-H); ^{13}C NMR (D_2O, 100 MHz) δ 8.58 (Ar-CH_3), 64.35 (COH), 113.39 (Ar-C), 119.87 (Ar-C), 142.60 (Ar-C), 145.46 (Ar-C), 174.54 (COOH); LRMS (ESI) *m/z*: Calculated for $C_7H_8O_4$ $[M + H]^+$: 157.1 Found: 157.0; Chiral-HPLC Retention Time: (*R*) enantiomer = 3.437 min. $[\alpha]_D^{25}$ = -52.6° (H_2O).

(R)-α-(4,5-dimethylfuran-2-yl)-α-hydroxyacetic acid **(9d)**, was prepared following the general procedure described above with **8d** (10 mg, 0.05 mmol), to give **9d** (7.1 mg, 89%) as a white solid. ^{1}H NMR (D_2O, 400 MHz) δ 1.78 (s, 3H; Ar-CH_3), 2.04 (s, 3H; Ar-CH_3), 5.12 (s, 1H; CH), 6.18 (s, 1H; Ar-H); ^{13}C NMR (D_2O, 100 MHz) δ 8.67 (Ar-CH_3), 10.27 (Ar-CH_3), 66.21 (COH), 112.67 (Ar-C), 115.14 (Ar-C), 147.33 (Ar-C), 148.84 (Ar-C), 174.19 (COOH); LRMS (ESI) *m/z*: Calculated for $C_8H_{10}O_4$ $[M + H]^+$: 170.1 Found: 170.0; Chiral-HPLC Retention Time: (*R*) enantiomer = 3.920 min. $[\alpha]_D^{25}$ = -78.4° (H_2O).

(R)-α-(5-methoxyfuran-2-yl)-α-hydroxyacetic acid **(9e)**, was prepared following the general procedure described above with **8c** (10 mg, 0.046 mmol), to give **9c** (isolated yield not determined) as a white solid. ^{1}H NMR (D_2O, 400 MHz) δ 3.75 (s, 3H; OCH_3), 4.80 (s, 1H; CH), 5.16 (d, J = 3.3 Hz, 1H; Ar-H), 6.20 (d, J = 3.3 Hz, 1H; Ar-H); ^{13}C NMR ($CDCl_3$, 100 MHz) δ 58.04 (OCH_3), 68.08 (COH), 80.35 (Ar-C), 110.54 (Ar-C), 142.84 (Ar-C), 161.16

(Ar-C), 176.86 (COOH); LRMS (ESI) *m/z*: Calculated for $C_7H_8O_5$ $[M + H]^+$: 172.1 Found: 172.0; Chiral-HPLC Retention Time: (*R*) enantiomer = 3.384 min. $[\alpha]_D^{25}$ = –55.5° (H_2O).

2-(Furan-2-yl) acetic acid **(13)**:[54] ^{1}H NMR ($CDCl_3$, 400 MHz) δ 3.34 (s, 2H; CH_2), 6.45 (m, 1H; Ar-H), 6.57 (d, J = 3.0 Hz, 1H; Ar-H), 7.46 (d, J = 1.5 Hz, 1H; Ar-H); ^{13}C NMR ($CDCl_3$, 100 MHz) δ 36.72 (CH_2), 107.10 (Ar-C), 110.66 (Ar-C), 141.94 (Ar-C), 151.04 (Ar-C), 178.56 (COOH); LRMS (ESI) *m/z*: Calculated for $C_6H_6O_3$ $[M + H]^+$: 126.1 Found: 127.0.

(S)-α-(thiophen-2-yl)-α-hydroxyacetic acid **(19)** was prepared following the general procedure described above with **18** (10 mg, 0.041 mmol), to give **19** (6.9 mg, 95%) as a white solid. ^{1}H NMR (D_2O, 400 MHz) δ 5.43 (s, 1H, C*H*OH), 7.20 (dd, J = 5.0, 3.5 Hz, 1H, Ar-H), 7.28 (d, J = 3.5 Hz, 1H, Ar-H), 7.58 (d, J = 5.0 Hz, 1H). ^{13}C NMR (101 MHz, D_2O) δ 70.66 (COH) 126.56 (Ar-H), 126.60 (Ar-H) 127.62 (Ar-H) 138.20 (Ar-H) 168.80 (COOH) IR (neat) : 3331, 1613, 1380, 1320, 1101 cm^{-1}. HR-MS (ES, m/z): Calculated for $C_6H_6O_3S$ $[M-H]^-$: 152.0353 Found: 152.0348. Chiral-HPLC Retention Times: (*R*) enantiomer = 3.774 min.

(R)-α-(benzofuran-2-yl)-α-hydroxyacetic acid **(23)** was prepared following the general procedure described above with **22** (10 mg, 0.036 mmol), to give **23** as a white solid (7.5 mg, 99%) ^{1}H NMR (D_2O, 400 MHz) δ 5.05 (s, 1H, HCOH), 6.73 (s, 1H, C3-H) 7.15 (m, 1H; C5-H), 7.21 (m, 1H; C6-H), 7.40 (d, J = 8.1 Hz, 1H; C7-H)), 7.51 (d, J = 7.7 Hz, 1H; C4-H) ^{13}C NMR (101 MHz, D_2O) δ 66.87 (COH), 106.03 (C3), 111.33 (C7), 121.72 (C4), 123.28 (C5), 125.22 (C6), 127.56 (C8), 153.21 (C2), 154.75 (C9) 173.70 (COOH) IR (neat): 3297, 1634, 396, 1330, 1053cm^{-1}. HR-MS (ES, m/z): Calculated for $C_{10}H_8O_4$ $[M-H]^-$: 191.0349 Found: 191.0356. Chiral-HPLC Retention Times: (*R*) enantiomer = 4.241 min. $[\alpha]_D^{25}$ = +25.0° (MeOH), Literature[S2] $[\alpha]_D^{25}$ = +26.0° (MeOH).

(R)-α-(pyridin-3-yl)-α-hydroxyacetic acid **(26)** was prepared following the general procedure described above with **25** (10 mg, 0.041 mmol), to give **26** (7.2 mg, 99%) as a white solid. ^{1}H NMR (D_2O, 400 MHz) δ 5.11 (s, 1H; C*H*OH), 7.51 (dd, J = 7.8, 5.4 Hz, 1H; C5-H), 7.91 (d, J = 7.8 Hz, 1H, C4-H), 8.52 (d, J = 5.4 Hz, 1H, C6-H), 8.59 (s, 1H, C2-H), ^{13}C NMR ($CDCl_3$, 101 MHz) δ 72.83 (COH), 124.63 (C5), 135.94 (C3), 137.08 (C4), 147.58 (C2), 148.44 (C6), 178.73 (COOH), IR (neat) : 3215, 1631, 1453, 1328, 1180 cm^{-1}. HR-MS (ES, m/z): Calculated for $C_7H_7O_3N$ $[M-H]^-$: 156.9964 Found: 156.9971. Chiral-HPLC Retention Times: (*R*) enantiomer = 7.669 min. $[\alpha]_D^{25}$ = –67.8° (H_2O), Literature[S4] $[\alpha]_D^{25}$= –65.2° (H_2O).

(R)-α-(benzo[b]thiophen-5-yl)-α-hydroxyacetic acid **(29)** was prepared following the general procedure described above with **28** (10 mg, 0.034 mmol), to **29** (7.3 mg, 94%) as a white

solid. ^{1}H NMR (400 MHz, D_2O) δ 5.00 (s, 1H; C*H*OH), 7.31 (d, *J* = 8.3 Hz, 1H; Ar-H), 7.36 (d, *J* = 4.2 Hz, 1H; Ar-H), 7.54 (d, *J* = 5.5 Hz, 1H; Ar-H), 7.82 (s, 1H), 7.88 (d, *J* = 8.3 Hz, 1H; Ar-H). ^{13}C NMR ($CDCl_3$, 101 MHz) δ 74.98 (COH), 122.27 (C3), 122.84 (C7), 123.19 (C2), 123.22 (C4), 124.00 (C6), 127.76 (C8), 136.96 (C5), 139.65 (C9), 179.51 (COOH). IR (neat) 3294, 1718, 1050, 747, 697 cm^{-1}. LR-MS (ES, m/z) Calculated for $C_{10}H_8O_3S$ $[M+Na]^+$ 231.0, Found: 231.0 Chiral-HPLC Retention Times: (*R*) enantiomer = 5.523 min. $[\alpha]_D^{25}$ = –131.0˚ (MeOH), Literature[S3] $[\alpha]_D^{25}$ (MeOH) = –142.3˚.

Calculation of kinetic parameters (K_m and k_{cat}): In order to calculate the kinetic parameters associated with a particular substrate, varied concentrations of the substrate were analysed using the colorimetric assay conditions described previously.[1,2] The change in absorbance (ΔA) at 620 nm for each substrate concentration was tracked over a specific time period (Δt) using a UV-Vis photospectrometer. The rate of decarboxylation (v) at each substrate concentration was then determined, allowing for the calculation of K_m and k_{cat} using standard Michaelis-Menten kinetics.

Calculation of enantiomeric excess values: Chiral HPLC was used to analyse the enantiomeric excess (*e.e.*) value associated with the decarboxylation of each substrate by AMDase, in comparison with racemic standards prepared from non-enzymatic decarboxylation of malonic acid substrates. In an Eppendorf tube, the substrate (2.5 mg/mL in 25 mM TRIS buffer at pH 7, 10 μL) was dissolved in TRIS buffer (25 mM at pH 7, 200 μL). The Eppendorf tube was then incubated at 37 °C for 15 minutes before the addition of AMDase (~0.001 mM in 0.154 g DTT, 1.89 g TRIS and 500 mL of distilled H_2O, pH 8, 1 μL). The solution was then incubated overnight at 37 °C. Subsequently, a 20 μL sample of this solution was loaded onto an Astec Chirobiotic column (5 μm particle size, 15 cm x 4.6 cm). The solvents used to achieve separation were (A) water (65%), methanol (25%) and 0.1% triethylammonium acetate (TEAA) (10%) and (B) methanol (100%) with the elution gradients formed as follows. For compounds **9a** to **9e** (A):(B) 100:0 from 0 to 15 min; 100:0 to 0:100 from 15 to 20 min; 0:100 from 20 to 25 min; 0:100 to 100:0 from 25 to 30 min; 100:0 from 30 to 45 min. For compounds **19**, **23**, **26** and **29** (A):(B) 100:0 from 0 to 15 min; 100:0 to 0:100 from 15 to 20 min; 0:100 from 20 to 25 min; 0:100 to 100:0 from 25 to 30 min; 100:0 from 30 to 45 min. The flow rate was 0.5 mL/min. UV detection was performed at 220 nm and/or 254 nm dependent on the compound involved.

Confirming the absolute configuration of AMDase produced α-hydroxy carboxylic acids using Mosher esters: The configuration of the enzymatically produced α-hydroxy carboxylic acids is based on the stereochemical course of AMDase catalysed decarboxylation reaction as determined previously by detailed labelling experiments and high resolution X-ray structures of AMDase (Fig. 1)[1-2] along with the configuration of many AMDase products as determined previously.[1-7] Comparison of optical rotations of the chiral products with literature $[\alpha]_D$ values where these are available (compounds **23**, **26** & **29**) are also consistent with the stated absolute (see page S12-S13). In addition the absolute configuration α-hydroxy carboxylic acids can be confirmed *via* the preparation of their respective Mosher esters[25], followed by a comparison of their TLC retention time with Mosher esters formed using racemic α-hydroxy carboxylic acids (Fig. S1). The general procedure used will be illustrated by the example of α-hydroxy-α-(thiophen-2-yl) acetic acid **19**.

Accordingly, racemic α-hydroxy-α-(thiophen-2-yl) acetic acid **19** was produced by dissolving the respective α-hydroxy-α-(thiophen-2-yl) malonic acid **18** (0.5 g, 0.00247 mol) in water (10 mL), which was then acidified to pH 1 using hydrochloric acid. This solution was then refluxed over the course of 3 hours, after which the solution was cooled to 0 °C and then neutralised using a concentrated NaOH solution. After removal of the solvent *in vacuo*, the racemic α-hydroxy-α-(thiophen-2-yl) acetic acid **19** (0.1 g, 0.00063 mol) was esterified by refluxing in ethanol (5 mL) in the presence of concentrated sulphuric acid (2 mL). After three hours, this reaction was neutralised using a concentrated NaOH solution, and the solvent was removed *in vacuo*. The racemic α-hydroxy-α-(thiophen-2-yl)-ethyl acetate **31** was recovered in quantitative yield following a chloroform/water extraction. The racemic α-hydroxy-α-(thiophen-2-yl) ethyl acetate **30** was then derivatised using (*R*)-α-methoxy-α-(trifluoromethyl)phenylacetyl chloride to produce the respective diastereoisomeric Mosher esters **31**.[25] To do this, α-(thiophen-2-yl)-α-hydroxy ethyl acetate **30** (0.023 g, 0.124 mmol) and N,N-dimethylaminopyridine (DMAP) (0.018 g, 0.15 mmol) were first dissolved in diethyl ether (5 mL) under anhydrous conditions. Under stirring, (*R*)-α-methoxy-α-(trifluoromethyl) phenylacetyl chloride (0.157 g, 0.62 mmol) was added to the solution in a dropwise manner, resulting in the immediate precipitation of DMAP. The reaction was stirred for a further hour, after which the reaction was diluted with diethyl ether (5 mL) and washed with brine (3x 2 mL). The organic extract was then dried over $MgSO_4$ and evaporated to yield the crude product **31** as an oil. The diastereoisomeric Mosher esters **31** were then

purified by preparative TLC using a 10:1 mixture of hexane and ethyl acetate as the eluent. This resulted in the resolution of two distinct bands after approximately 4 hours. These bands were cut out from the TLC plate, and each band was seperately submerged in methanol to recover the purified diastereoisomer. This solution was then filtered to remove any excess silica, before the methanol was removed *in vacuo*. Each sample was then characterised using ^{1}H NMR, with the difference in the position of the heterocyclic signals between the diastereoisomers being used to determine the stereochemistry of the C_{α}-O bond. This ^{1}H NMR analysis showed that the top band isolated *via* TLC was (*R*)-(*S*) configured and the bottom band was (*S*)-(*S*) configured (Table S1).

Mosher esters were similarly prepared from AMDase produced enantiopure α-hydroxy-α-(thiophen-2-yl) acetic acid **19**; subsequent comparison of the products from this process with that of the racemic process showed that the enzymatic products are (*R*)-configured (Fig. S1).

α-Hydroxy-α-(thiophen-2-yl) ethyl acetate **(30)**: ^{1}H NMR (400 MHz, $CDCl_3$) δ 1.23 (t, *J* = 7.1 Hz, 3H), 4.29 – 4.15 (m, 2H), 5.33 (s, 1H), 6.93 (dd, *J* = 5.1, 3.5 Hz, 1H), 7.04 (dt, *J* = 3.5, 1.1 Hz, 1H), 7.22 (dd, *J* = 5.1, 1.1 Hz, 1H). ^{13}C NMR (101 MHz, $CDCl_3$) δ 14.06, 62.61, 69.07, 125.31, 125.67, 126.95, 141.53, 172.50.

(2S)-2-ethoxy-2-oxo-1-(thiophen-2-yl)ethyl-3,3,3-trifluoro-2-methoxy-2-phenylpropanoate **(31)**: HR-MS (ES, m/z): Calculated for $C_{18}H_{17}O_5SF_3$ $[M+NH_4]^+$: 420.1087 Found: 420.1079. For ^{1}H NMR see table S1.

Circular dichroism (CD) spectroscopy. To further support the configurational assignments, CD spectra of the α-heteroaryl-α-hydroxyacetic acids were acquired and compared with CD spectra of (*S*)-**19** and an authentic natural sample of (*R*)-mandelic acid (these two compounds are of the same homochiral series and only differ in stereochemical descriptor due to a change in the CIP priority). Far ultraviolet (UV) CD spectra for products of the AMDase reaction were recorded on a Chirascan CD spectrometer (Applied Photophysics Limited, U.K) at 20°C. Compounds were dissolved in acetonitrile to 0.006mol/L, and measured using a 1 nm spectral bandwidth, step size 0.5 nm, and a cell path length of 1 mm. The CD spectra (Figs. S2-S10) all exhibit similar Cotton effects with a positive band at shorter wavelength, below 190 nm, and a negative band at longer wavelengths typically between 210-250nm. The CD spectra are also consistent with spectra reported previously[S5, S6] for (*S*)-**19** and (*R*)-mandelic acid, as well as other mandelic acid derivatives. These literature precedents,[S5, S6] also indicate that the sign of the Cotton effects in the CD spectra of α-aryl-α-hydroxyacetic acids are comparable across a homochiral series.

References for the Supporting Information.

[S1] A. Oussaid, F. Benyaqad and B. Oussaid *et. al.*, *Phosphorous, Sulfur and Silicon* **2003**, *178*, 1605-1616.

[S2] M. A. Naghi, L. C. Bencze, J. Brem, C. Paizs, F. D. Irimie and M. Tosa, *Tetrahedron Asymmetry* **2012**, *23*, 181-187.

[S3] J. Nakano, N. Taya, H. Chaki and T. Yamafuji, *Benzo[b]thiophen-5-yl derivative and process for producing the same* **1993**, European Patent: 0 565 965 A2.

[S4] G. Desantis, Z. Zhu, W. A. Greenberg, K. Wong, J. Chaplin, S. R. Hanson, B. Farwell, L. W. Nicholson, C. L. Rand, D. Weiner, D. Robertson and M. J. Burk, *J. Am. Chem. Soc.* **2002**, *124*, 9024-9025.

[S5] R. Håkansson, S. Gronowitz, *Tetrahedron* 1976, *32*, 2973-2976.

[S6] O. Korver *Tetrahedron* 1970, *26*, 5507-5518.

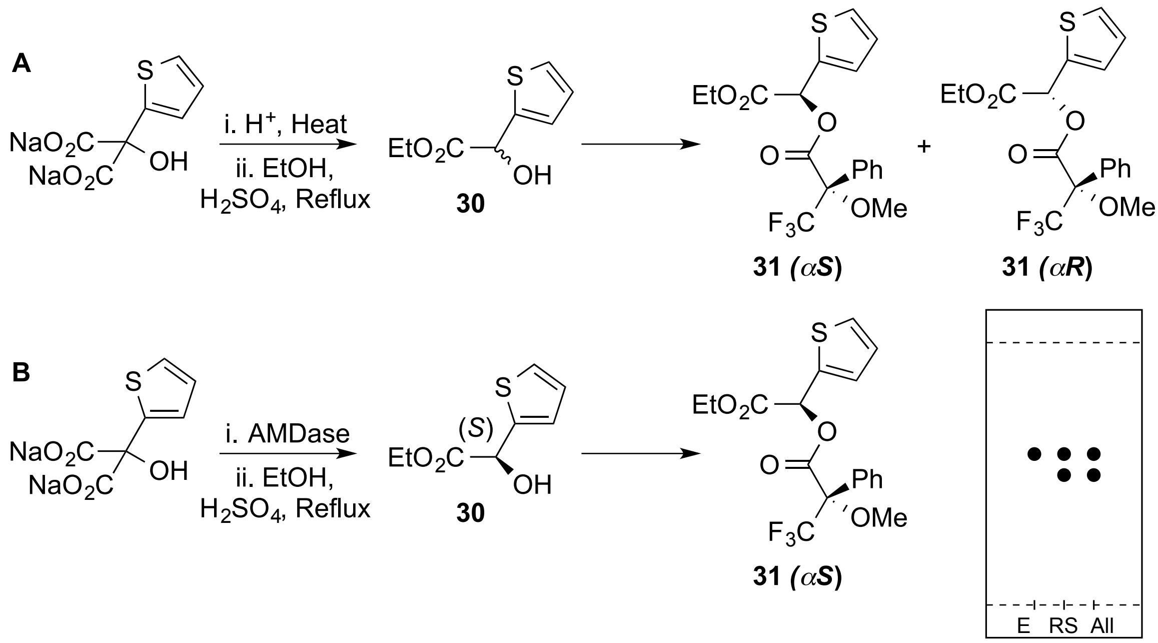

Figure S1: Mosher esters were produced from both (A) non-enzymatic decarboxylation of **18** to give a mixture of diastereoisomers **31**(α*R* and α*S*) and (B) enzymatically to give a single diateroisomer **31**(α*S*). Subsequent separation of the diastereoisomeric Mosher esters performed *via* preparative TLC allowed for the stereospecificity of each band to be assigned using ^{1}H NMR. From there, determination of the R_F value for enzymatically produced Mosher esters allowed for the determination of the stereochemical course of AMDase catalysed decarboxylation reactions.

^{1}H NMR Peak	Top Band (Higher R_F) δ (ppm)	Bottom Band (Lower R_F) δ (ppm)	Difference δ (ppm)
Central Proton (α)	6.257	6.303	0.046
Thiophenyl Triplet (2)	6.919	6.951	0.032
Thiophenyl Doublet (3)	7.075	7.132	0.057
Thiophenyl Doublet (1)	7.280	7.310	0.030

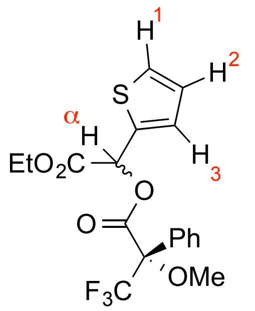

Table S1: ^{1}H NMR parameters used to determine the absolute configuration of 2-ethoxy-2-oxo-1-(thiophen-2-yl)ethyl 3,3,3-trifluoro-2-methoxy-2-phenylpropanoate diastereoisomers separated *via* preparative TLC. The data is consistent with the top band (the enzymatic product) containing the (α*S*) configured diastereoisomer and the bottom band containing the (α*R*) configured diastereoisomer.

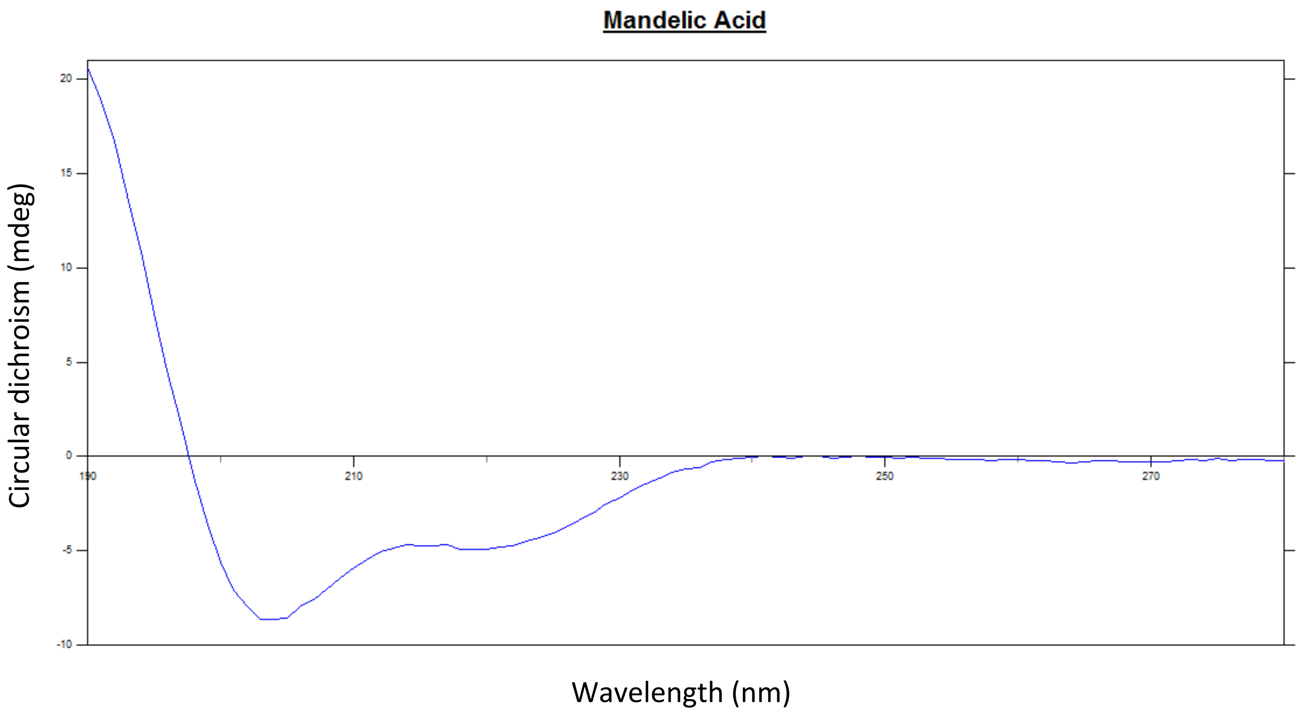

Figure S2. CD spectrum of (*R*)-Mandelic acid

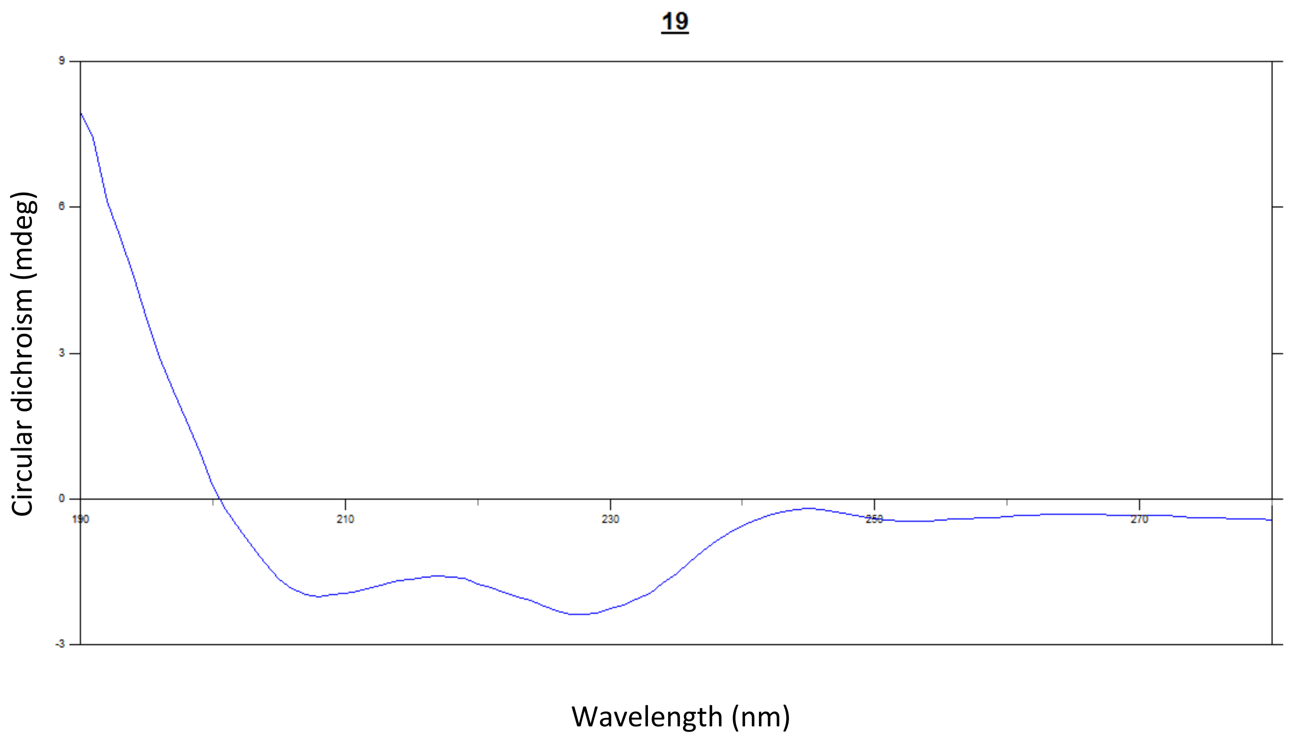

Figure S3. CD spectrum of (*S*)-α-(thiophen-2-yl)-α-hydroxyacetic acid **(19)**

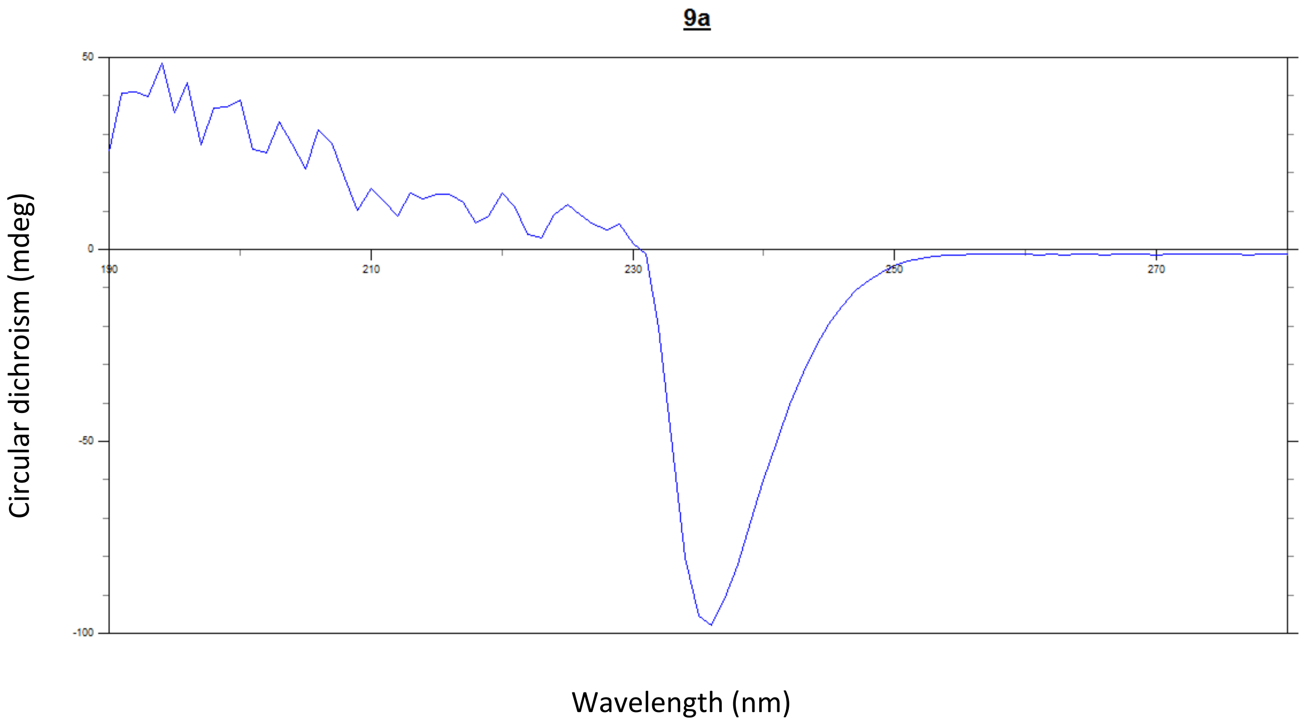

Figure S4. CD spectrum of (*R*)-α-(furan-2-yl)-α-hydroxyacetic acid (**9a**)

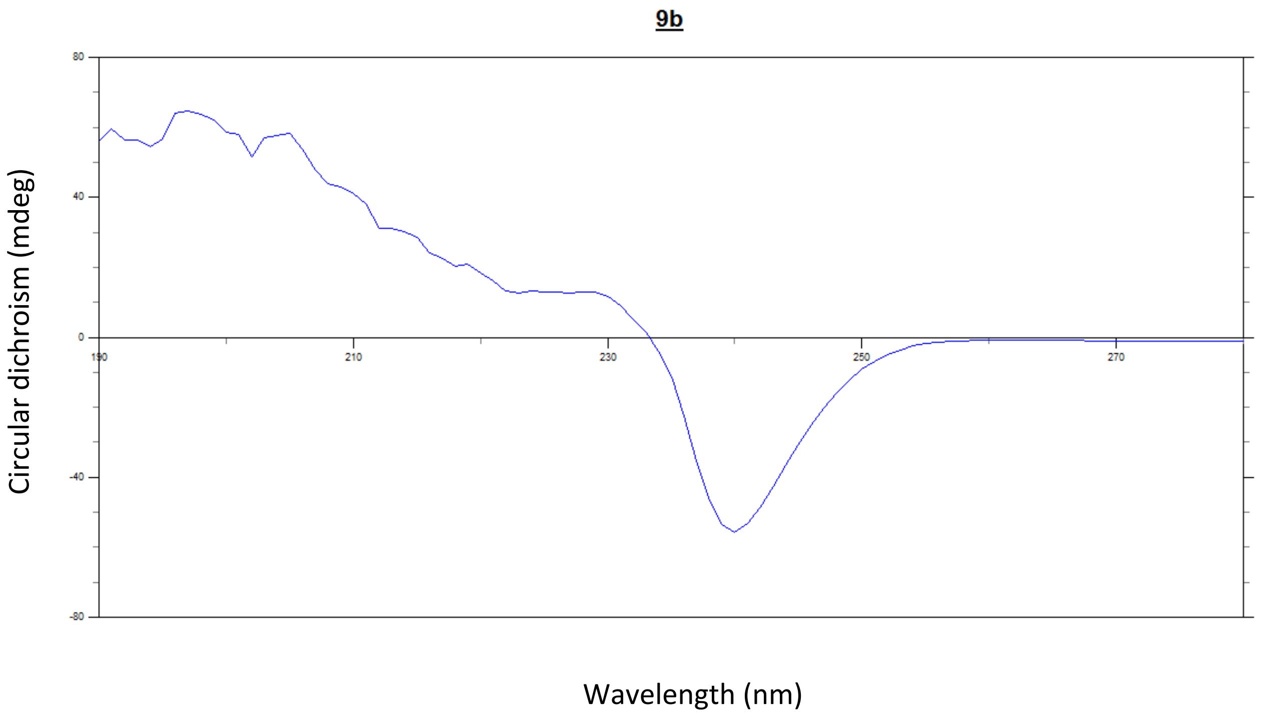

Figure S5. CD spectrum of (*R*)-α-(5-methylfuran-2-yl)-α-hydroxyacetic acid **(9b)**

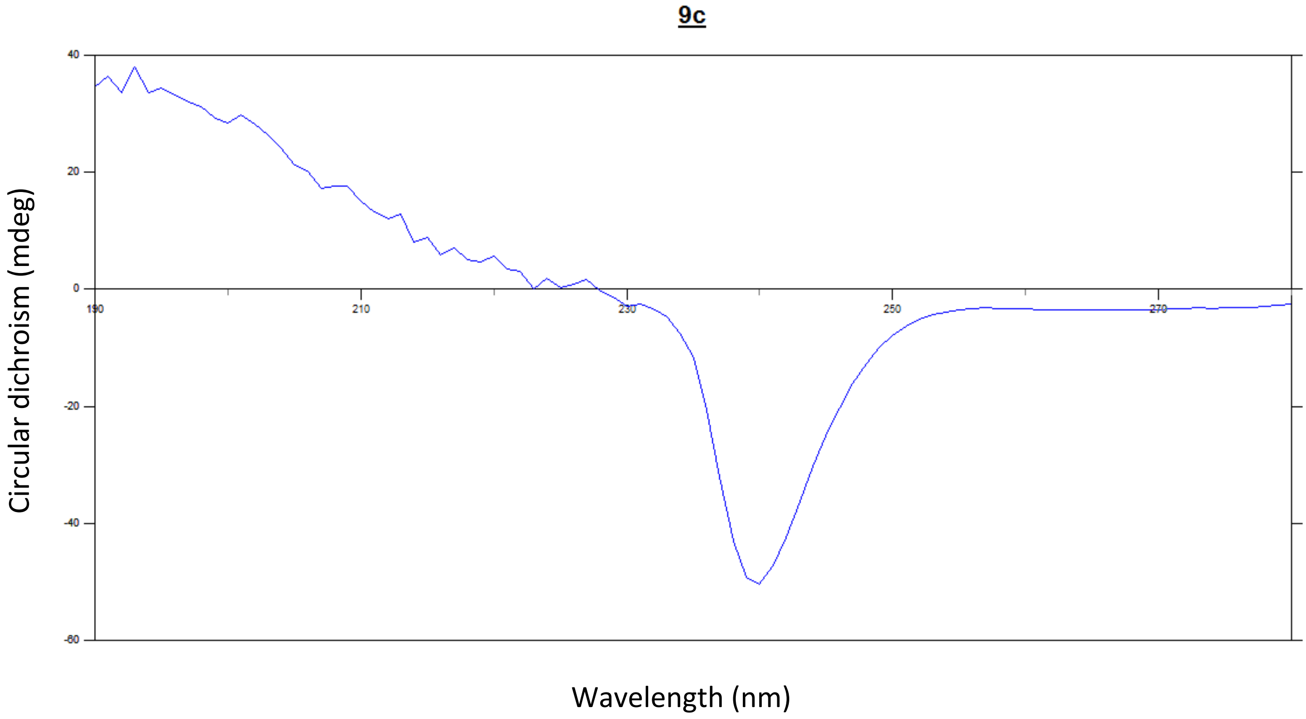

Figure S6. CD spectrum of (*R*)-α-(4-methylfuran-2-yl)-α-hydroxyacetic acid **(9c)**

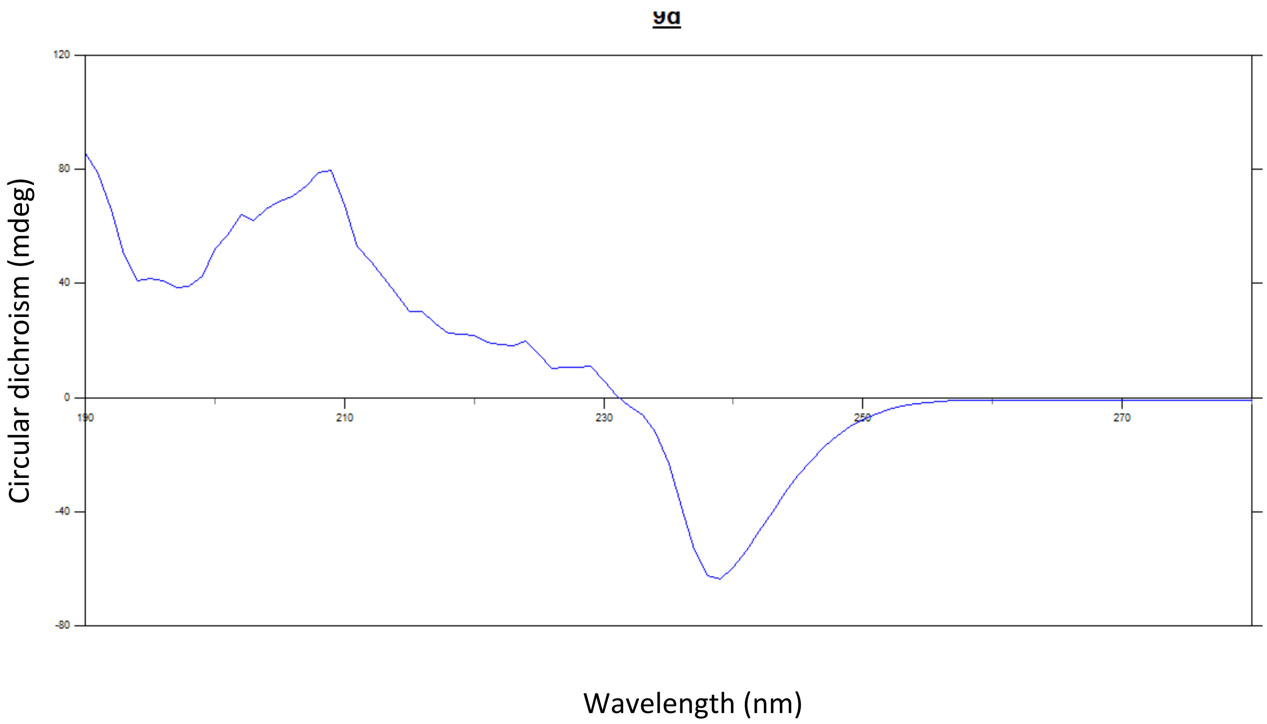

Figure S7. CD spectrum of (*R*)-α-(4,5-dimethylfuran-2-yl)-α-hydroxyacetic acid **(9d)**

23

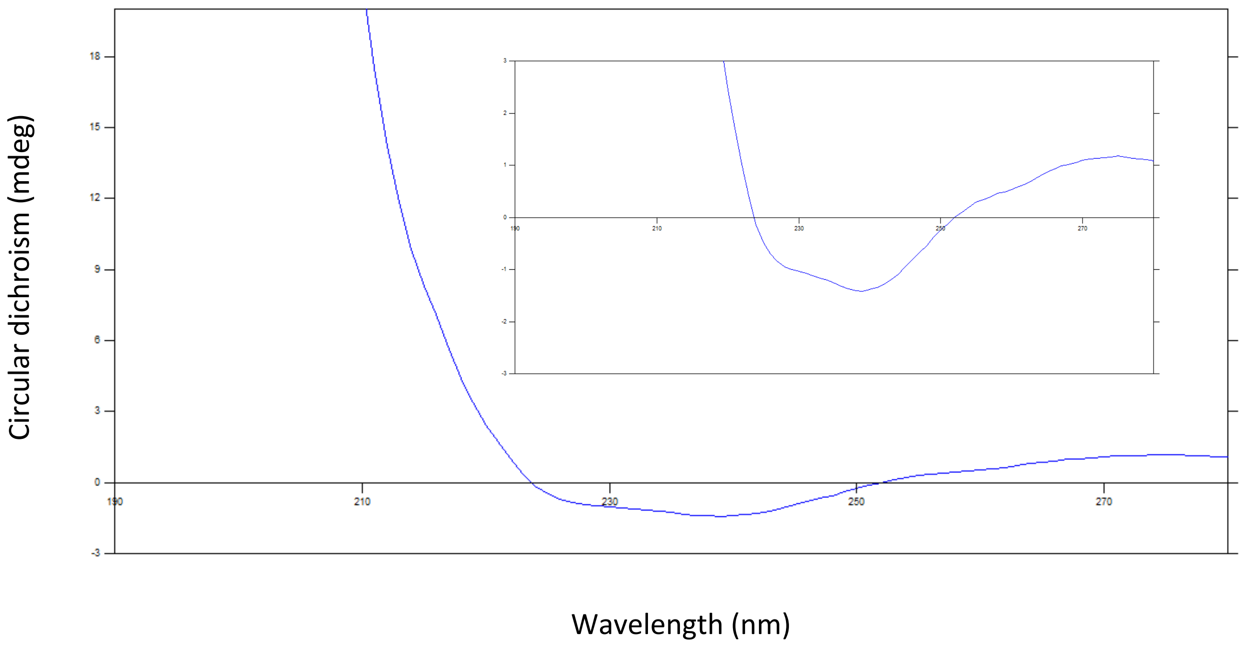

Figure S8. (*R*)-α-(benzofuran-2-yl)-α-hydroxyacetic acid **(23)**

26

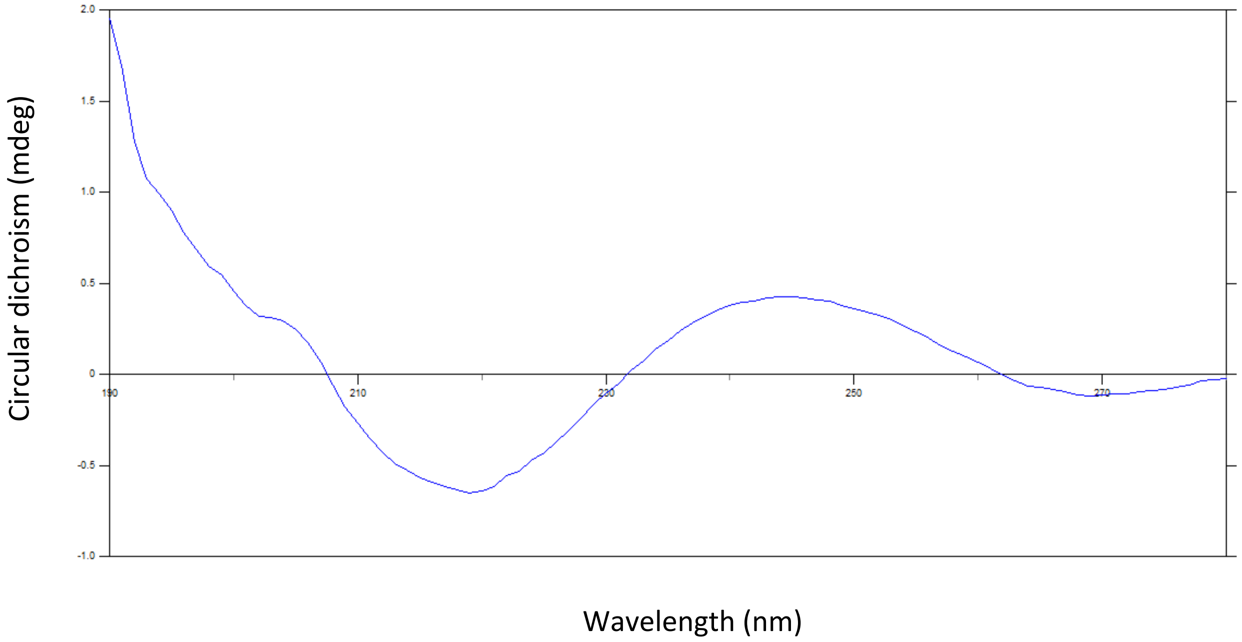

Figure S9. CD spectrum of (*R*)-α-(pyridin-3-yl)-α-hydroxyacetic acid **(26)**

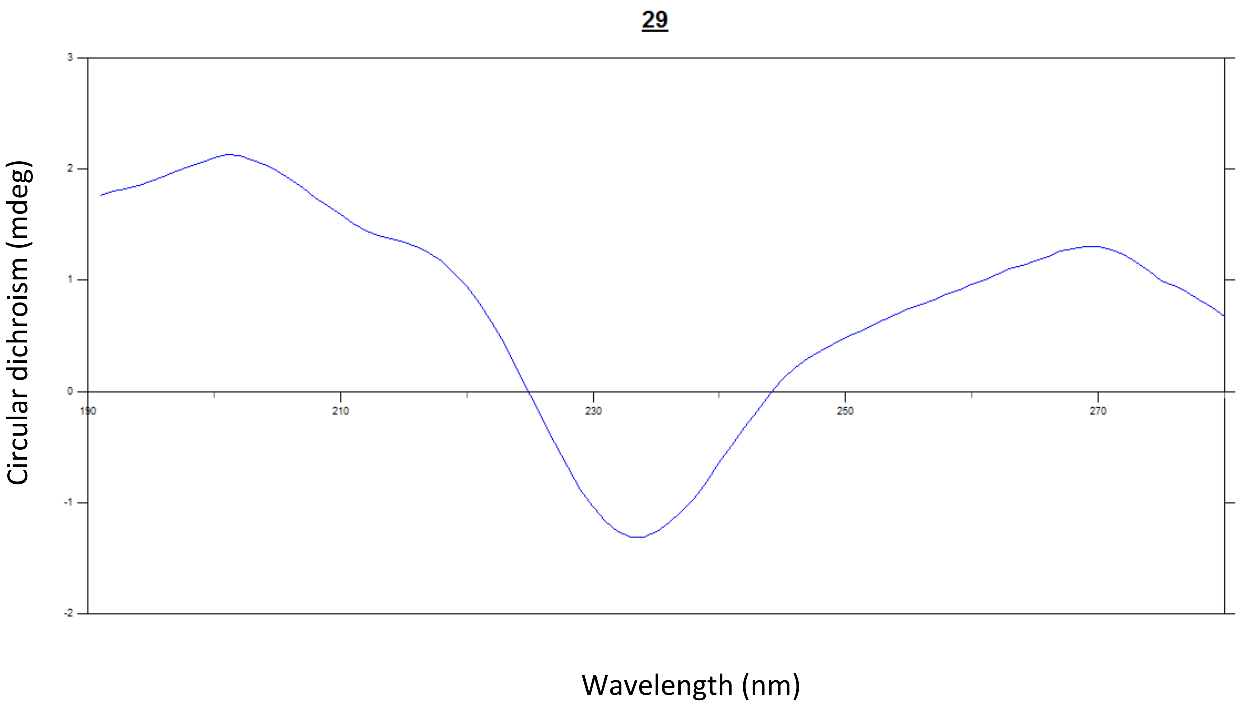

Figure S10. (*R*)-α-(benzo[b]thiophen-5-yl)-α-hydroxyacetic acid
